# OTUD7B Deubiquitinates LSD1 to Govern Its Binding Partner Specificity, Homeostasis, and Breast Cancer Metastasis

**DOI:** 10.1002/advs.202004504

**Published:** 2021-05-29

**Authors:** Zhicheng Gong, Aicun Li, Jiancheng Ding, Qing Li, Lei Zhang, Yuanpei Li, Zhe Meng, Fei Chen, Jialiang Huang, Dawang Zhou, Ronggui Hu, Jing Ye, Wen Liu, Han You

**Affiliations:** ^1^ State Key Laboratory of Cellular Stress Biology Innovation Center for Cell Signaling Network School of Life Sciences Xiamen University Xiamen Fujian 361102 China; ^2^ School of Pharmaceutical Sciences Fujian Provincial Key Laboratory of Innovative Drug Target Research Xiamen University Xiamen Fujian 361102 China; ^3^ State Key Laboratory of Molecular Biology Shanghai Science Research Center CAS Center for Excellence in Molecular Cell Science Shanghai Institute of Biochemistry and Cell Biology Chinese Academy of Sciences University of Chinese Academy of Sciences Shanghai 200031 China; ^4^ Department of Pathology Xijing Hospital Fourth Military Medical University Xi'an Shanxi 710032 China

**Keywords:** deubiquitination, epigenetic modification, gene transcription, metastasis

## Abstract

Genomic amplification of OTUD7B is frequently found across human cancers. But its role in tumorigenesis is poorly understood. Lysine‐specific demethylase 1 (LSD1) is known to execute epigenetic regulation by forming corepressor complex with CoREST/histone deacetylases (HDACs). However, the molecular mechanisms by which cells maintain LSD1/CoREST complex integrity are unknown. Here, it is reported that LSD1 protein undergoes K63‐linked polyubiquitination. OTUD7B is responsible for LSD1 deubiquitination at K226/277 residues, resulting in dynamic control of LSD1 binding partner specificity and cellular homeostasis. OTUD7B deficiency increases K63‐linked ubiquitination of LSD1, which disrupts LSD1/CoREST complex formation and targets LSD1 for p62‐mediated proteolysis. Consequently, OTUD7B deficiency impairs genome‐wide LSD1 occupancy and enhances the methylation of H3K4/H3K9, therefore profoundly impacting global gene expression and abrogating breast cancer metastasis. Moreover, physiological fluctuation of OTUD7B modulates cell cycle‐dependent LSD1 oscillation, ensuring the G1/S transition. Both OTUD7B and LSD1 proteins are overpresented in high‐grade or metastatic human breast cancer, while dysregulation of either protein is associated with poor survival and metastasis. Thus, OTUD7B plays a unique partner‐switching role in maintaining the integrity of LSD1/CoREST corepressor complex, LSD1 turnover, and breast cancer metastasis.

## Graphical abstract

OTUD7B catalyzes K63‐linked deubiquitination of lysine‐specific demethylase 1 (LSD1), a process that is pivotal in regulating cellular homeostasis and the binding partner specificities of LSD1. OTUD7B regulates LSD1‐dependent transcriptome via modulating genome‐wide LSD1 distribution and H3K4me2 enrichment. Both proteins are overpresented in high‐grade or metastatic human breast cancer, highlighting a unique role of dysregulated OTUD7B signaling in driving breast cancer metastasis via LSD1.

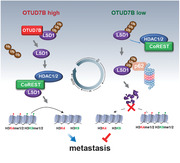

## 1 Introduction

Lysine‐specific demethylase 1 (LSD1) is known to act on gene transcription, via demethylating both histone and nonhistone substrates, to regulate diverse biological functions.^[^ ^1^ ^]^ Emerging evidence has shown that the histone demethylase function of LSD1 relies on its association with multiple factors, including CoREST and histone deacetylases (HDACs) 1 and 2.^[^ ^2^, ^3^, ^4^ ^]^ Meanwhile, LSD1 has been found to undergo phosphorylation,^[^ ^5^ ^]^ acetylation,^[^ ^6^ ^]^ and ubiquitination,^[^ ^7^, ^8^, ^9^ ^]^ resulting in distinct biological outcomes. However, the post‐translational modifications of LSD1 that may regulate the assembly and dynamics of LSD1/CoREST/HDACs corepressor complex remain completely unknown. Moreover, the influence of the known post‐translational modifications on LSD1‐dependent transcriptome at the global scale remains unclear.

A number of studies have suggested a crucial role of LSD1 in cell proliferation^[^ ^1^ ^]^ Interestingly, LSD1 oscillates during the cell cycle, with protein levels peaking in G2/M,^[^ ^10^ ^]^ but the molecular mechanism underlying this physiological turnover of LSD1 remains unexplored. Deubiquitination has been shown to govern LSD1 stability.^[^ ^7^, ^8^, ^9^ ^]^ Three deubiquitinases have been reported to regulate LSD1 steady state, namely, USP22,^[^ ^7^ ^]^ USP28,^[^ ^8^ ^]^ and USP7.^[^ ^9^ ^]^ However, the underlying mechanistic details of LSD1 deubiquitination, as well as its impact on corepressor complex formation have not been investigated.

Here, we found OTUD7B, a deubiquitinase belonging to the ovarian tumor (OTU) family of DUBs,^[^ ^11^ ^]^ is responsible for LSD1 deubiquitination. OTUD7B has been reported to regulate cell cycle, tumorigenesis, neural progenitor cell differentiation, inflammatory responses, mucosal immunity, and diseases associated with the noncanonical NF‐kB pathway by catalyzing deubiquitination of substrates including Cyclin B, Aurora A, epidermal growth factor receptor (EGFR), G*β*L, Sox2, Zap70, and TRAF3.^[^ ^12^, ^13^, ^14^, ^15^ ^]^ Genomic amplification of OTUD7B is frequently found across human cancers. But the role of this deubiquitinase in tumorigenesis is poorly understood. Our data unraveled a pivotal role of OTUD7B in gene transcription, cell proliferation, and cancer metastasis by modulating LSD1 stability and its assembly into corepressor complex.

## 2 Results

### 2.1 OTUD7B Binds LSD1 and Regulates Its Stability

Aberrant expression of LSD1 has been extensively reported in human cancers. However, examination of genetic alterations and transcript level of *LSD1* gene from 38 cancer types containing approximate 10 000 samples from The Cancer Genome Atlas (TCGA) database revealed overall very low incidence of mutation rate, low frequency of loss or gains of *LSD1*, and negligible changes of its mRNA levels across most cancers (Figure S1A,B and Tables S1 and S2, Supporting Information), suggesting dysregulated LSD1 signaling occurs largely through post‐translational modifications of this demethylase.^[^ ^16^ ^]^

**Figure 1:**
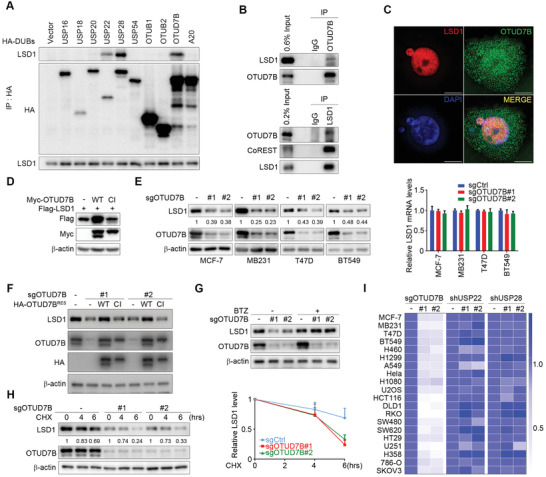
OTUD7B binds LSD1 and regulates its stability. A) HA‐tagged DUBs were transfected into HEK293T cells, followed by immunoprecipitation (IP) and immunoblotting (IB) as indicated. B) Co‐IP of OTUD7B with LSD1 in MDA‐MB‐231 cells. The immunoprecipitated materials by the indicated antibodies were analyzed by western blotting. C) Colocalization of the indicated proteins by IF staining with the indicated antibodies was visualized by structured illumination microscopy (SIM). Scale bars: 10 µm. D) Flag‐tagged LSD1 was co‐transfected into HEK293T cells with the indicated constructs. Whole cell lysates (WCLs) were subjected to IB analysis. E) Cells were infected with the indicated lentiviruses. Cell lysates and RNA extracts were subjected to IB (left panel) and quantitative real‐time PCR (qRT‐PCR) analysis as indicated (right panel). F) OTUD7B reconstituting MDA‐MB‐231 cells were subjected to IB analysis as indicated. G) MDA‐MB‐231 cells stably expressing the indicated sgRNAs were treated with BTZ (100 × 10^−9^ m) for 5 h, followed by IB analysis. H) MDA‐MB‐231 cells stably expressing OTUD7B sgRNAs were treated with cycloheximide (CHX, 50 µg mL^−1^), followed by IB analysis as indicated (left panel). The graph shows the quantification of protein levels (right panel). I) IB analysis of WCL derived from 20 different cancer cells expressing the indicated lentiviral constructs, as shown in (E) and Figures S1J and S2A in the Supporting Information. The heatmap shows the fold change of quantified LSD1 protein levels. Data were presented as mean ± SEM of three independent experiments.

Previous reports found USP22 and USP28 regulate LSD1 stability. However, ubiquitin (Ub) linkage specificity, the responsible lysine residues, and most importantly, the physiological conditions that link the deubiquitination process of LSD1 to its proteolysis, remain completely unknown. To gain more insights into the physiological regulation of LSD1 ubiquitination, we screened a DUB library that consists of 100 known or putative DUBs. In addition to USP22 and USP28, ectopic OTUD7B also bound robustly to endogenous LSD1 (**Figure** **1A**). Of note, both wild type (WT) and the catalytically inactive form (CI, C194A/H358R) of OTUD7B bound ectopic LSD1 (Figure S1C,D, Supporting Information). Interestingly, when compared to LSD1–CoREST complex, endogenous OTUD7B–LSD1 complex is readily detectable but with much less abundance, indicating OTUD7B may interact with LSD1 in a transient manner (Figure 1B). The nuclear colocalization of OTUD7B and LSD1 was further verified by immunofluorescent (IF) staining (Figure 1C). Notably, we were unable to detect any significant interaction between OTUD7B and CoREST when both were ectopically expressed, even in cells expressing ectopic LSD1 (Figure S1E, Supporting Information). Using recombinant proteins, we concluded that OTUD7B can directly interact with LSD1 in vitro (Figure S1F, Supporting Information). Mapping the regions of interaction revealed that OTUD7B associated with the N‐terminals of LSD1, whereas the UBA and ZNF regions of OTUD7B were required for LSD1 binding (Figure S1G,I, Supporting Information).

We noticed that ectopically expressed WT OTUD7B significantly increased Flag‐LSD1 levels in HEK293T cells, whereas the CI mutant failed to do so (Figure 1D). Conversely, OTUD7B depletion in several breast cancer cell lines profoundly decreased LSD1 protein level, without affecting *LSD1* mRNA expression (Figure 1E). We next generated OTUD7B reconstituting cell lines by expressing sgRNA‐resistant WT or mutant OTUD7B construct in MDA‐MB‐231 cells depleted of OTUD7B, and found only WT OTUD7B reconstitution rescued LSD1 decrease, excluding a possible off‐target issue during sgRNA‐mediated knockdown, also suggesting the DUB catalytic function of OTUD7B is required for its regulation of LSD1 (Figure 1F). Bortezomib (BTZ), a proteasome inhibitor, did restore the reduced LSD1 levels upon OTUD7B knockdown (Figure 1G). We also observed shortened half‐life of LSD1 in the absence of OTUD7B (Figure 1H), indicating OTUD7B regulates LSD1 steady state via blocking its proteasome‐mediated degradation. By contrast, USP22 or USP28 silencing showed no effect on LSD1 turnover in breast cancer cell lines tested (Figure S1J, Supporting Information). Of note, shRNA constructs targeting USP22 or USP28 were generated using exact sequences from original research articles to avoid any potential biases. The above results prompted us to examine if OTUD7B‐mediated LSD1 stabilization is a common mechanism among tumor cell lines of different origins. Of 16 additional cancer cell lines tested, all lines exhibited significant LSD1 decrease when OTUD7B was depleted. In contrast, ablation of USP22 or USP28 only exhibited very marginal effects, if any, on LSD1 protein levels (Figure 1I; Figure S2A, Supporting Information). In addition, we confirmed that OTUD7B‐mediated LSD1 turnover occurred exclusively in the nucleus (Figure S2B, Supporting Information). Taken all together, these results suggest that OTUD7B binds and stabilizes LSD1 via abrogating its ubiquitination‐dependent proteasomal degradation.

### 2.2 OTUD7B Is a Bona Fide LSD1 Deubiquitinase

Ubiquitination of LSD1 has emerged as an important regulatory mechanism for its turnover.^[^ ^7^, ^8^, ^17^ ^]^ To determine the Ub‐linkage specificity, we transfected HEK293T cells with Myc‐tagged WT Ub or one of its variants with a single Lys‐Arg substitution of the seven lysine residues, namely, K6R, K11R, K27R, K29R, K33R, K48R, and K63R. K0 is an Ub mutant that all lysine residues are substituted with arginine. As shown in Figure S3A in the Supporting Information, K63R mutation caused significant loss of polyubiquitinated LSD1 signals, whereas K48R displayed very marginal reduction in LSD1 ubiquitination, suggesting LSD1 ubiquitination involves mixed linkage specificity, and predominantly prefers K63‐linked polyubiquitin chains.

**Figure 2:**
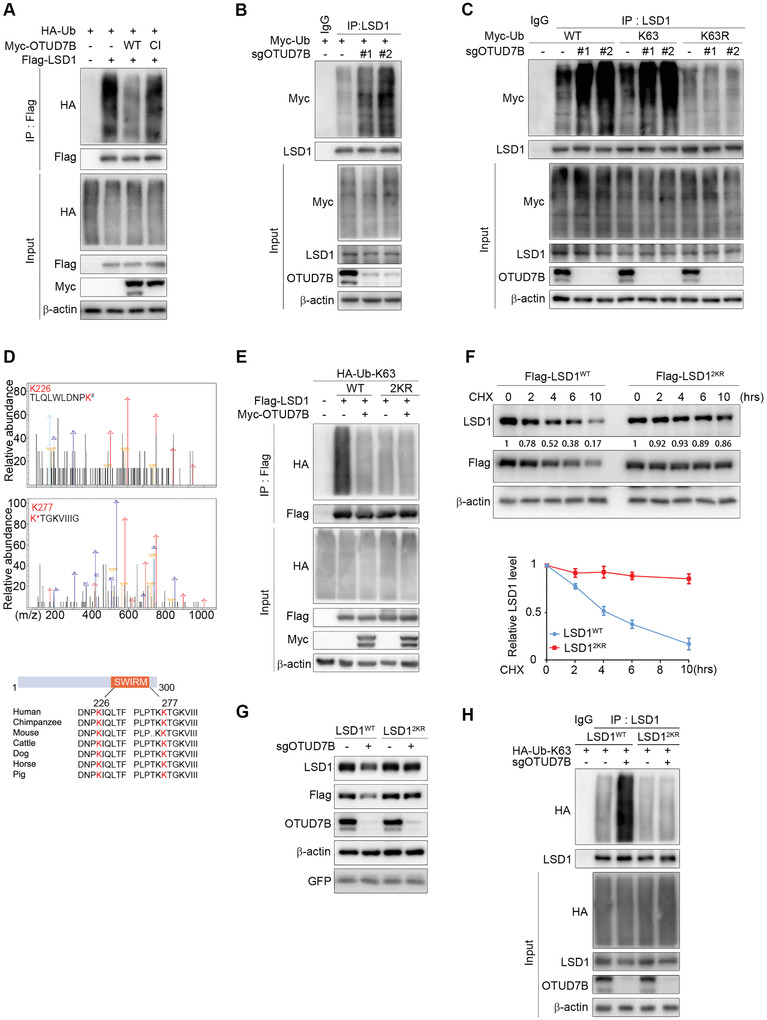
OTUD7B is a bona fide LSD1 deubiquitinase. A) IB analysis of WCL and Flag immunoprecipitate derived from HEK293T cells transfected with the indicated constructs and treated with BTZ for 5 h before harvesting. B) HEK293T cells stably expressing OTUD7B sgRNAs were transfected with Myc‐tagged Ub, followed by IP and IB analysis as indicated. C) HEK293T cells stably expressing OTUD7B sgRNAs were co‐transfected with the indicated Ub constructs, followed by IP and IB analysis as indicated. D) HEK293T cells were co‐transfected with Flag‐tagged LSD1, HA‐tagged ubiquitin, and Myc‐tagged OTUD7B. Cell lysates were subjected to IP and mass spectrometry analysis to identify ubiquitination sites. The recovered LSD1 peptide and the ubiquitination sites were highlighted in red (upper panel). Lower panel: A schematic diagram showing two evolutionarily conserved lysine residues (K226 and K277) within the SWIRM domain of LSD1. E) HEK293T cells were co‐transfected with the indicated plasmids, followed by IP and IB analysis as indicated. F) LSD1^WT^‐ or LSD1^2KR^‐reconstituted MDA‐MB‐231 cells were treated with CHX for the indicated times. Lysates were subjected to IB analysis as indicated (top panel). The graph shows the quantification of protein levels (bottom panel). G) IB analysis of WCL derived from the indicated LSD1 reconstituting cells expressing OTUD7B sgRNA lentiviruses. H) LSD1‐reconstituted cells stably expressing the indicated sgRNAs were transfected with K63‐only Ub plasmid, followed by IP and IB analysis as indicated. Data were presented as mean ± SEM of three independent experiments.

We next determined whether OTUD7B modulated LSD1 deubiquitination. When co‐transfected into 293T cells, only WT OTUD7B catalyzed ectopic LSD1 deubiquitination (**Figure** **2A**). Conversely, OTUD7B depletion remarkably enhanced polyubiquitination of endogenous LSD1 (Figure 2B). In vitro deubiquitination assay further confirmed that recombinant OTUD7B could remove the ubiquitin chain from LSD1 (Figure S3B, Supporting Information). By contrast, USP28 or USP22 only catalyzed the deubiquitination of ectopic LSD1 upon overexpression (Figure S3C, Supporting Information), whereas knockdown these DUBs failed to enhance the ubiquitination of endogenous LSD1 in our experiments (Figure S3D, Supporting Information).

To assess the types of Ub‐linkage that OTUD7B may catalyze when deubiquitinates LSD1, we utilized two Ub mutants, K48 and K63. Ectopic HA‐OTUD7B profoundly reduced the levels of K63‐linked, but not K48‐linked Flag‐LSD1 ubiquitination (Figure S3E, Supporting Information). Conversely, OTUD7B deficiency only enhanced K63‐linked polyubiquitination of endogenous LSD1 (Figure S3F, Supporting Information). In cells expressing K63R Ub mutant, OTUD7B‐depletion failed to increase LSD1 ubiquitination (Figure 2C). Together, these data demonstrate that OTUD7B is a bona fide LSD1 deubiquitinase that removes K63‐linked polyubiquitinated chain from LSD1.

Using Mass Spectrometry analysis, we attempted to map the site(s) on LSD1 for OTUD7B‐mediated deubiquitination. Lys226 and Lys277, two conserved lysine residues among different species (Figure 2D), appeared to be the major ubiquitination sites subjected to OTUD7B. To further confirm the role of these two lysines in LSD1 ubiquitination, we constructed dual substitution mutant‐LSD1^2KR^ (K226R/K277R) by replacing lysine with arginine. Compared to LSD1^WT^, LSD1^2KR^ conferred almost complete resistance to deubiquitination catalyzed by ectopic OTUD7B (Figure 2E), and the basal levels of polyubiquitinated LSD1^2KR^ were largely diminished as well. Furthermore, loss of the polyubiquitinated LSD1^2KR^ band in both the K63R and K48R mutant Ub experiments argues that LSD1^2KR^ indeed is ubiquitination‐deficient under basal conditions (Figure S3G, Supporting Information). To explore the physiological role for K63‐linked poly‐Ub chains in regulating LSD1, we generated LSD1‐reconstituted stable cell lines by depleting endogenous LSD1 and then reintroducing either Flag‐LSD1^WT^ or Flag‐LSD1^2KR^ constructs. Expressions of both LSD1^WT^ and LSD1^2KR^ in reconstituting cells were comparable to endogenous LSD1 levels (Figure S3H, Supporting Information). Intriguingly, LSD1^2KR^ not only exhibited significantly extended half‐life compared to LSD1^WT^ (Figure 2F), but also conferred resistance to OTUD7B depletion‐induced polyubiquitination and degradation (Figure 2G,H). These findings establish a critical role of OTUD7B in controlling LSD1 steady state through removing K63‐linked poly‐Ub chains on K226 and K277 residues of LSD1.

Previous study^[^ ^4^ ^]^ revealed a conspicuous surface groove formed by the interface between the AOD and the SWIRM domains of LSD1, and mutations of the residues within this groove impaired the demethylase activity of LSD1. Although K226 and K277 lie in the SWIRM and AOD domains, respectively, LSD1^2KR^ did not alter the enzymatic activity of LSD1 (Figure S3I, Supporting Information), whereas LSD1^K661A^, a demethylase defective mutant, displayed abrogated activity toward a histone substrate.

### 2.3 OTUD7B‐Mediated Removal of K63‐Linked Poly‐Ub Chains on LSD1 Determines LSD1/CoREST/HDACs Integrity

**Figure 3:**
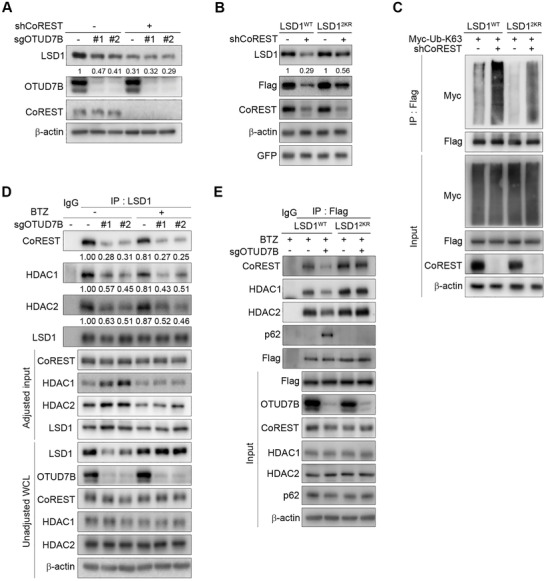
OTUD7B‐mediated removal of K63‐linked poly‐Ub chains on LSD1 determines LSD1/CoREST/HDACs integrity. A) MDA‐MB‐231 cells stably expressing the indicated lentiviruses were subjected to IB analysis. B) LSD1 reconstituting cells were infected with the indicated lentiviruses, followed by IB analysis. C) LSD1 reconstituting cells expressing CoREST shRNA were transfected with K63‐only Ub plasmid, followed by IP and IB analysis as indicated. D) MDA‐MB‐231 cells expressing OTUD7B sgRNAs were treated with BTZ for 5 h, followed by IP and IB analysis as indicated. E) IP and IB analysis of LSD1 reconstituting cells expressing OTUD7B sgRNA as indicated.

As a key binding partner of LSD1, CoREST regulates LSD1 stability^[^ ^3^ ^]^ by an unknown mechanism. Given the above observed regulatory role of OTUD7B in stabilizing LSD1, we sought to determine whether OTUD7B and CoREST are functionally interconnected in modulating LSD1 turnover. We first asked if CoREST is required for OTUD7B‐mediated LSD1 stabilization. As described earlier, in OTUD7B‐dificient cells, WT OTUD7B reconstitution fully restored LSD1 protein expression. However, this rescue effect was completely blunted when endogenous CoREST was absent (Figure S4A, Supporting Information), suggesting these two molecules might utilize similar mechanism regulating LSD1 steady state. This hypothesis was further supported by a combined knockdown experiment, where LSD1 destabilization triggered by CoREST deficiency alone failed to show further reduction upon simultaneous depletion of both CoREST and OTUD7B (**Figure** **3A**). Collectively, these results lead to our hypothesis that CoREST and OTUD7B regulates LSD1 stability via the same mechanism, specifically, through modulating K63‐linked LSD1 ubiquitination.

To test this possibility, we examined the type of poly‐Ub chains conjugated onto LSD1 in response to CoREST silencing. Similar to OTUD7B depletion, loss of CoREST led to profoundly increased K63‐linked polyubiquitination of LSD1 (Figure S4B, Supporting Information). However, in LSD1‐reconstituting cells, LSD1^2KR^ displayed only partial yet significant resistance to CoREST depletion‐induced destabilization (Figure 3B). Consistent with these observations, CoREST deficiency in LSD1^2KR^ reconstituting cells was still capable of promoting K63‐linked ubiquitination of LSD1, albeit to a much lesser extent compared to that in LSD1^WT^ cells (Figure 3C). These data raised the possibility that CoREST might modulate LSD1 stability through preventing K63‐linked LSD1 polyubiquitination, on K226/277 and possibly some other yet unidentified residues.

Protein ubiquitination often modulates diverse cellular processes. We speculated that, in addition to regulating its proteolytic degradation, K63‐linked polyubiquitination of LSD1 may modulate its association with the CoREST/HDACs complex. To test this, we examined the endogenous LSD1/CoREST/HDACs complex abundance in cells depleted of OTUD7B. BTZ treatment blocked LSD1 degradation, without restoring the amount of LSD1/CoREST/HDACs complex in OTUD7B‐deficient cells (Figure 3D), suggesting K63‐linked ubiquitination at K226/K277 residues indeed abrogated LSD1 association with these binding partners. We also examined LSD1/CoREST complex in LSD1 reconstituting cells. Unlike LSD1^WT^, LSD1^2KR^ exhibited very steady binding capacity toward CoREST/HDACs, disregarding the presence or absence of OTUD7B (Figure 3E). These results suggested a causal link between OTUD7B‐mediated LSD1 deubiquitination and its binding affinity to CoREST/HDACs complex.

### 2.4 K63‐Linked Polyubiquitination of LSD1 Facilitates Its Proteasomal Degradation via Promoting Interaction with p62

K63‐linked Ub chains usually have nonproteolytic functions.^[^ ^18^ ^]^ Having shown that OTUD7B‐mediated deubiquitination controlled LSD1 turnover, we next focused on elucidating the molecular mechanisms underlying LSD1 degradation. Stable isotope labeling with amino acids in cell culture (SILAC)‐based quantitative proteomics analyses were performed to uncover potential binding partners involved in LSD1 proteolysis. Among all hits identified in LSD1 coimmunoprecipitates, p62, a polyubiquitin chain binding protein, turned out to be one of the binding partners based on a normalized SILAC ratio (Figure S4C, Supporting Information).

**Figure 4:**
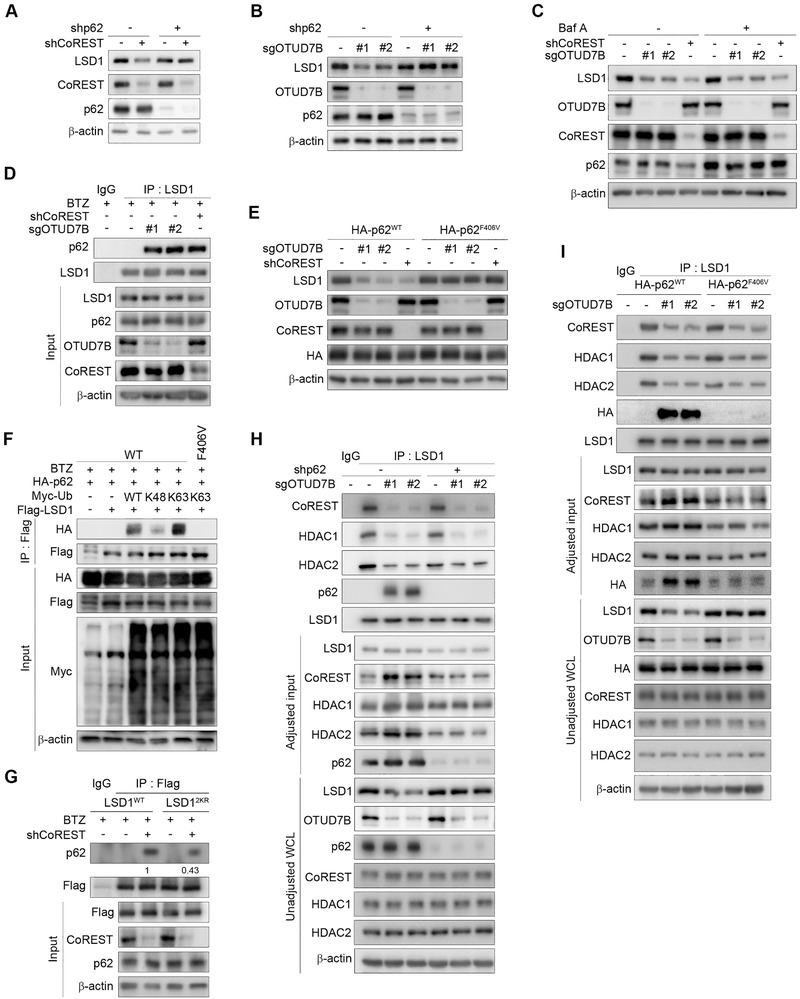
K63‐linked polyubiquitination of LSD1 facilitates its proteasomal degradation via promoting interaction with p62. MDA‐MB‐231 cells stably expressing A) CoREST shRNA or B) OTUD7B sgRNAs were infected with lentiviruses encoding p62 shRNA, followed by western blotting. C) MDA‐MB‐231 cells stably expressing CoREST shRNA or OTUD7B sgRNAs were treated with Baf A (200 × 10^−9^ m) for 6 h followed by IB analysis. D) IP and IB analysis of MDA‐MB‐231 cells infected with the indicated lentiviral constructs. E) p62 reconstituting MDA‐MB‐231 cells expressing the indicated lentiviruses were subjected to IB analysis. F) HEK293T cells transfected with the indicated plasmids were collected for IP with anti‐Flag antibody, followed by IB analysis as indicated. G) LSD1 reconstituting cells infected with the indicated lentiviral constructs were collected for IP and IB analysis. H) MDA‐MB‐231 cells stably expressing the indicated lentiviruses were subjected to IP and IB analysis. I) p62 reconstituting cells infected with the indicated lentiviruses were collected for IP and IB analysis. Data were presented as mean ± SEM of three independent experiments.

p62 has been shown to preferentially recognize K63‐linked poly‐Ub chains, therefore shuttling substrates for proteasomal degradation.^[^ ^19^ ^]^ Strikingly, p62 knockdown completely blunted LSD1 degradation triggered by OTUD7B or CoREST loss (**Figure** **4A**,B). By contrast, ablating lysosomal degradation machinery by pharmacologic inhibition failed to rescue LSD1 deduction upon OTUD7B or CoREST silencing (Figure 4C). These data demonstrated that p62 is critically required for the proteolytic degradation of LSD1.

Interestingly, knockdown OTUD7B or CoREST seemed to significantly promote the formation of p62–LSD1 complex (Figure 4D). To obtain mechanistic insights into p62‐mediated LSD1 degradation, we generated p62 reconstituting cells by depleting endogenous p62, followed by adding back shRNA‐resistant p62^WT^ or p62^F406V^ (a mutant defected in ubiquitin chain binding ^[^ ^20^ ^]^). OTUD7B or CoREST depletion only resulted in LSD1 degradation in p62^WT^ cells, but not in p62^F406V^ cells (Figure 4E), suggesting that the binding to poly‐Ub chains on LSD1 is indispensable for p62‐mediated LSD1 degradation triggered by the deficiency of OTUD7B or CoREST.

We next determined the type of poly‐Ub chains predisposed LSD1 to p62 binding. As shown in Figure 4F, K63‐linked ubiquitination of LSD1 exhibited much stronger binding affinity to p62, indicating a crucial role for K63‐linked poly‐Ub chains in facilitating LSD1–p62 complex formation. As expected, only WT p62 associated with K63‐linked LSD1, further underscoring the importance of K63‐linked polyubiquitination on promoting LSD1 interaction with p62. Consistently, in LSD1 reconstituting cells, OTUD7B deficiency only promoted p62 interaction with LSD1^WT^, but not with LSD1^2KR^ (Figure 3E). Similar results were obtained in LSD1‐reconstituting cells upon CoREST depletion (Figure 4G). Notably, in CoREST‐depleted cells, LSD1^2KR^ markedly reduced, but did not completely abolish its binding to p62 compared to LSD1^WT^, in agreement with the possibility that additional lysine sites are subjected to K63‐linked ubiquitination when CoREST is absent (Figure 4G). Collectively, these results demonstrate that K63‐linked ubiquitination of LSD1, triggered by depletion of OTUD7B or CoREST, targets LSD1 to proteasomal degradation via p62.

The enhanced interaction between p62 and LSD1 upon OTUD7B loss raised one possibility that p62 may compete with CoREST for binding to ubiquitinated LSD1. Therefore, the simultaneously diminished corepressor complex formation might be secondary to the reduced pool of LSD1, which was available for CoREST binding. To clarify this, we first depleted p62 in OTUD7B deficient cells. In these cells, the amount of LSD1 associating with CoREST/HDACs was still profoundly reduced, similar to cells expressing control shRNA (Figure 4H). Using p62^F406V^ reconstituting cells, we further confirmed the binding ability of LSD1 toward CoREST/HDACs was largely reduced in OTUD7B‐deficient cells, despite the presence or absence of functional p62 (Figure 4I). These data again highlight a critical physiological role for K63‐linked poly‐Ub chains in determining the corepressor complex integrity.

### 2.5 OTUD7B‐Dependent Regulation of LSD1 Fluctuation Is Crucial for Cell Cycle Progression

**Figure 5:**
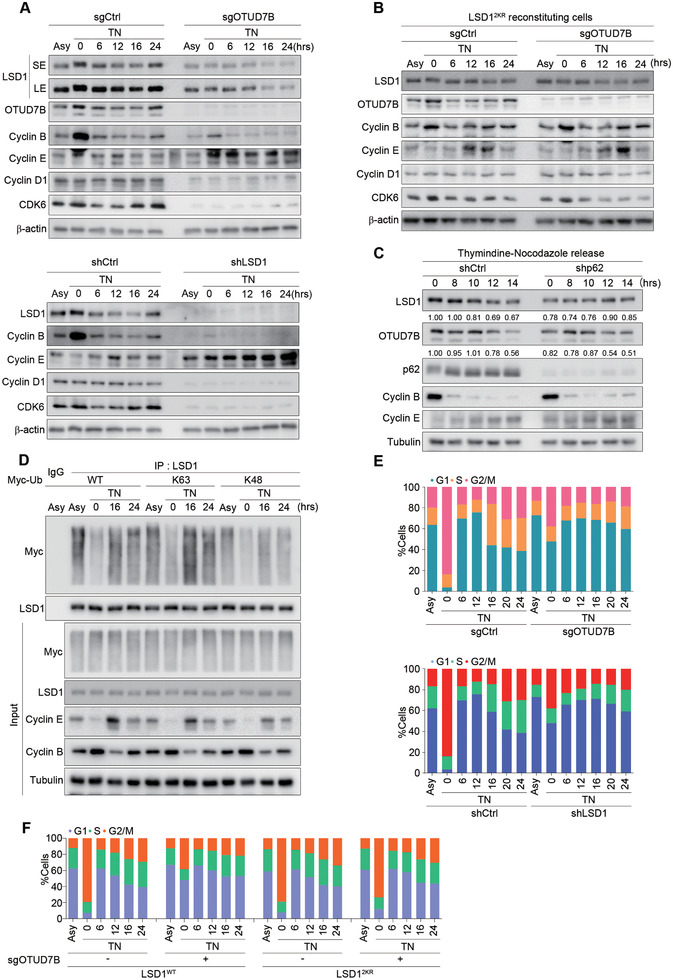
OTUD7B‐dependent regulation of LSD1 fluctuation is crucial for cell cycle progression. A) MDA‐MB‐231 cells stably expressing either OTUD7B sgRNA (top panel) or LSD1 shRNA (bottom panel) were synchronized by thymidine (2 × 10^−6^ m) and nocodazole (200 ng mL^−1^) treatment, released and harvested at the indicated time points. Cell lysates were subjected to IB analysis as indicated. Asy: asynchronous cells. SE: shorter exposure; LE: longer exposure. B) LSD1^2KR^‐reconstituting cells infected with sgOTUD7B lentiviruses were synchronized and released as in (A), followed by IB analysis. C) MDA‐MB‐231 cells infected with lentiviral p62 shRNA were synchronized as in (A), released and harvested at the indicated times, followed by IB analysis. D) MDA‐MB‐231 cells transfected with the indicated Ub constructs were synchronized as in (A) and released. Cell lysates were subjected to IP and IB analysis as indicated. E,F) Parental or LSD1‐reconstituting MDA‐MB‐231 cells infected with the indicated lentiviral constructs were synchronized as in (A), released and collected at the indicated time points. Cell cycle profile was obtained by propidium iodide (PI) staining and fluorescent‐activated cell sorting (FACS) analysis. TN: thymidine and nocodazole treatment. Data shown are representative of three independent experiments.

LSD1 protein expression oscillates during cell cycle, low in G1 and high in G2/M.^[^ ^10^ ^]^ Similar expression patterns were observed with OTUD7B.^[^ ^12^ ^]^ We therefore set out to investigate if OTUD7B–p62 axis is responsible for LSD1 ubiquitination and degradation throughout the cell cycle. Using MDA‐MB‐231 cells synchronized in M phase and then released into cell cycle from a thymidine/nocodazole block, we confirmed the oscillation of LSD1 and OTUD7B (Figure S5A, Supporting Information). Notably, protein levels of CoREST, p62, USP22, or USP28 remain unchanged along the time. To determine if OTUD7B is responsible for periodic fluctuation of LSD1 expression, we synchronized OTUD7B‐depleted cells and found OTUD7B silencing completely blunted the dynamic LSD1 fluctuation as cells progressed into cell cycle (**Figure** **5A**). Furthermore, unlike levels of LSD^WT^ that exhibited fluctuating expression pattern (Figure S5B, Supporting Information), LSD1^2KR^ remained unchanged throughout the cell cycle (Figure 5B). These data strongly support the physiological impact of OTUD7B‐mediated LSD1 deubiquitination on its cell cycle‐dependent fluctuation. Importantly, p62 depletion completely blocked LSD1 decrease following release into G1 phase (Figure 5C). Taken all together, these results establish a crucial role of K63‐linked LSD1 ubiquitination in regulating its turnover via p62 during cell cycle progression.

We speculated that K63‐linked LSD1 ubiquitination may also oscillate during the cell cycle. Indeed, K63‐linked polyubiquitinated LSD1 was greatly reduced in cells synchronized in G2/M, but markedly increased once cells entered G1 phase (16 h after releasing) (Figure 5D). Similar results were obtained using cells expressing WT‐Ub construct. These data strongly suggest that the fluctuation in K63‐linked polyubiquitination of LSD1 dynamically controls its stability throughout the cell cycle.

Given the dynamic and strict regulation of LSD1 by OTUD7B during cell cycle progression, we next asked if OTUD7B depletion may influence cell cycle. Cells with OTUD7B or LSD1 depletion failed to arrest at G2/M following synchronization. Instead, majority cells were arrested at G1 (Figure 5E; Figure S5C, Supporting Information). We next examined cell cycle distribution upon OTUD7B knockdown in LSD1 reconstituting cells. Compared to LSD1^WT^ reconstitution, LSD1^2KR^ exhibited resistance to OTUD7B deficiency‐induced G1 arrest (Figure 5F; Figure S5D, Supporting Information), suggesting K63‐linked polyubiquitination of LSD1 at K226/277 is a determinant in G1/S transition.

### 2.6 OTUD7B Regulates Gene Transcription in a LSD1‐Dependent Manner

Since OTUD7B regulates the integrity of LSD1/CoREST corepressor complex, we speculated OTUD7B may influence genomic occupancy of LSD1 and enrichment of H3K4me2‐modified chromatin. To test this, we first sought to gain a global view of the chromatin distribution pattern of LSD1 by ChIP sequencing (ChIP‐seq). Consistent with previous reports,^[^ ^21^ ^]^ majority of LSD1‐binding signal was found to be enriched at introns (37%) or intragenic regions (38%) in MDA‐MB‐231 cells (hereinafter termed enhancers) (Figure S6A, Supporting Information). About 16% (6069) of 43 872 LSD1 peaks showed dramatic loss of LSD1 signals upon OTUD7B knockdown (fold change (FC) > 2, *P* < 10^−4^) (Figure S6B, Supporting Information), suggesting a crucial role of OTUD7B in modulating overall genome‐wide LSD1 binding.

LSD1 is known to catalyze both histone H3K4 and K9 demethylation. Due to lack of a reliable anti‐H3K9me2 antibody, we only performed H3K4me2 ChIP‐seq. About 40% of H3K4me2 peaks were localized at promoters, whereas 58% were detected at enhancers (Figure S6A, Supporting Information). Among them, 3672 H3K4me2 peaks showed significant increase upon OTUD7B loss (FC > 2, *P* < 10^−4^) (Figure S6B, Supporting Information). Further analysis identified 1067 OTUD7B‐dependent H3K4me2 peaks occurred in regions lost LSD1 binding (Figure S6C, Supporting Information), 21% were localized to promoters (*P* = 3.56 × 10^−66^), and 77% were enriched at distal enhancer regions (*P* = 6.32 × 10^−150^) (Figure S6D,E, Supporting Information), suggesting OTUD7B loss‐induced H3K4me2 gains occurred mainly at enhancers, consistent with altered LSD1 distribution pattern.

**Figure 6:**
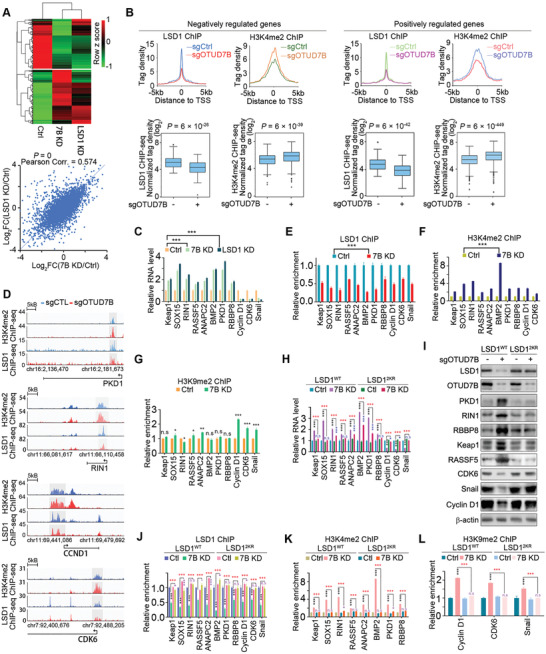
OTUD7B regulates gene transcription in an LSD1‐dependent manner. A) Heatmap of differentially expressed (FDR < 0.05) genes among control, OTUD7B KD, or LSD1 depleted MDA‐MB‐231 cells (top panel). Correlation analysis of differentially expressed genes in OTUD7B or LSD1 KD normalized to control (bottom panel). *P* values were calculated by Fisher's exact test. B) Normalized LSD1 and H3K4me2 tag densities at promoters of OLco‐regulated targets under control or OTUD7B depleted condition (top panel). Box plots displaying the change of LSD1 or H3K4me2 tag density induced by OTUD7B loss (bottom panel). *P* values were calculated by Student's *t* test. C) qRT‐PCR results show fold change of mRNA levels of the indicated OLco‐regulated genes in MDA‐MB‐231 cells infected with the indicated lentiviruses. D) Examples of LSD1 and H3K4me2 ChIP‐seq tracks surrounding the promoters of the indicated genes upon OTUD7B knockdown. ChIP assay was performed with the indicated antibodies, followed by qRT‐PCR analysis of fold enrichment of E) LSD1, F) H3K4me2, and G) H3K9me2 at promoter regions of the indicated genes. LSD1 reconstituting cells infected with the indicated lentiviruses were collected and subjected to H) qRT‐PCR and I) western blotting analysis as indicated. LSD1 reconstituting cells infected with the indicated lentiviruses were subjected to ChIP as indicated, followed by qRT‐PCR analysis of the enrichment of J) LSD1, K) H3K4me2, and L) H3K9me2 at promoter regions of the indicated genes. ^*^ *P* < 0.05, ^**^ *P* < 0.01, and ^***^ *P* < 0.001. *P* values were determined by two‐tailed unpaired *t* test. Data were presented as mean ± SEM of three independent experiments.

We then performed RNA‐seq analysis in MDA‐MB‐231 cells depleted of either OTUD7B or LSD1 (**Figure** **6A**; Figure S6F, Supporting Information). A total of 1308 negatively regulated and 1120 positively regulated genes were identified as overlapping set of targets between LSD1‐ and OTUD7B‐regulated transcriptome (herein defined as OLco‐regulated genes) (Figure S6G, Supporting Information). Pathway analysis of these overlapping genes enriched “cell cycle” and “cell division” among the most significant categories (Figure S6H, Supporting Information). The fact that many of these gene hits are linked to cell cycle likely explains the result that the primary functions of OTUD7B or LSD1 in coordinating cell cycle progression.

The above ChIP‐seq and RNA‐seq datasets were aligned to explore the potential correlations between OTUD7B‐induced H3K4me2 and LSD1 occupancy changes and changes in gene transcription. Notably, among OLco‐regulated genes, reduced LSD1 recruitment upon OTUD7B knockdown was mainly observed at enhancer regions (Figure S6I, Supporting Information), whereas gain of H3K4me2 was more pronounced at promoters (Figure S6J, Supporting Information). To assess potential mechanistic clues explaining biological outcomes triggered by dysregulated OTUD7B/LSD1‐axis, we focused on genes whose promoters lost LSD1 occupancy and gained H3K4me2 following OTUD7B depletion. Using this criteria, 258 OLco‐negatively and 274 OLco‐positively regulated genes were identified, respectively (Figure 6B).

A total of 11 OLco‐regulated genes with functions related to “tumorigenesis and cell cycle regulation,” including 8 negatively regulated (*Keap1*, *Sox15*, *RIN1*, *RASSF5*, *ANAPC2*, *BMP2*, *PKD1*, and *RBBP8*) and 3 positively regulated (*CyclinD1*, *CDK6*, and *Snail*), were selected for further studies. Among them, *BMP2* and *Cyclin D1* have been reported by other groups as LSD1‐regulated genes.^[^ ^22^, ^23^ ^]^ Expression levels of these genes and their proteins were verified by quantitative real‐time PCR (qRT‐PCR) and western blotting using OTUD7B‐ or LSD1‐depleted cells (Figure 6C; Figure S6K, Supporting Information). Consistent with ChIP‐seq results (Figure 6D), significant loss of LSD1 binding signals and accumulation of H3K4me2 marks at promoters of these 11 genes were observed by quantitative ChIP (ChIP‐qPCR) upon OTUD7B depletion (Figure 6E,F). We speculate that the OLco‐positively regulated genes may simultaneously gain H3K9me2 repressive mark, which ultimately caused gene transrepression upon OTUD7B or LSD1 loss. Indeed, ChIP‐qPCR assay using H3K9me2 antibody detected sharply increased H3K9me2 signals at *Cyclin D1*, *CDK6*, and *snail* promoters (Figure 6G). Therefore, perturbation of OTUD7B–LSD1 axis results in changes in both activating and repressive chromatin modification marks, providing a plausible mechanism for altered gene transcription due to OTUD7B loss.

To investigate the contribution of LSD1 deubiquitination by OTUD7B in OTUD7B‐mediated LSD1 genomic distribution and H3K4me2/H3K9me2 enrichment, we analyzed LSD1 binding and H3K4me2/H3K9me2 levels of the selected 11 genes in LSD1 reconstituting cells. Validation of their mRNA levels revealed that, compared to LSD1^WT^ reconstitution, LSD1^2KR^ conferred resistance to OTUD7B depletion‐induced alteration of their transcripts expression (Figure 6H). Also, for these genes, changes in their protein expression patterns were quite consistent with those at transcriptional levels (Figure 6I). Importantly, in LSD1‐reconstituted cells, LSD1^2KR^ rendered their promoters resistant to OTUD7B ablation‐induced H3K4me2/H3K9me2 alterations (Figure 6J,L). These results highlight a pivotal role of OTUD7B‐mediated deubiquitination of LSD1 in modulating its genomic occupancy and the regulation of gene transcription.

### 2.7 OTUD7B Promotes Metastasis via LSD1

**Figure 7:**
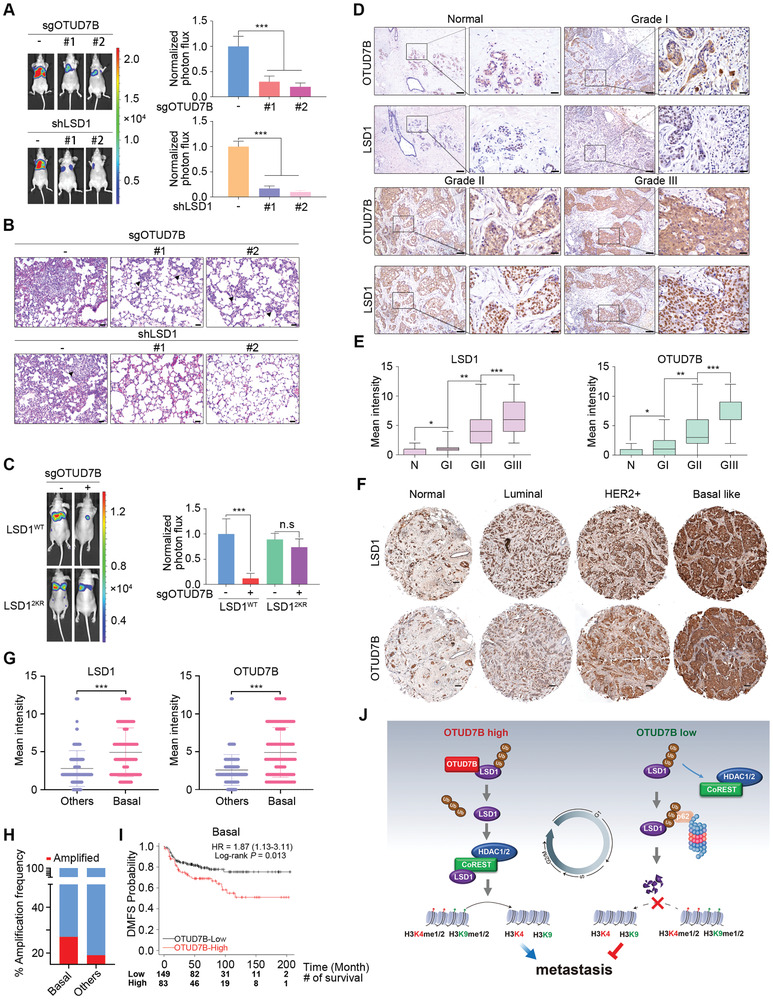
OTUD7B promotes metastasis via deubiquitinating LSD1. A) Parental or C) LSD1 reconstituting LM2 cells infected with the indicated lentiviruses were injected into the mammary fat pads of nude mice. Representative bioluminescent images of mice with spontaneous lung metastasis (left panel), quantification (right panel), and (B) representative H&E staining analysis of lung metastasis is shown. Results represent mean ± SEM. n = 8 mice per group. Scale bars: 40 µm. ^***^P < 0.001. *P* values were calculated by two‐tailed unpaired *t* test. D) Representative IHC staining of LSD1 and OTUD7B in normal breast tissues (*n* = 26) and breast carcinomas (*n* = 379) (histological I, II, and III). Scale bars: 100 µm for low magnification (10×) and 25 µm for high magnification (40×). E) Quantification of LSD1 and OTUD7B intensity in (D). *P* values were calculated by two‐tailed unpaired *t* test. ^*^ *P* < 0.05, ^**^ *P* < 0.01, and ^***^ *P* < 0.001. F) Representative IHC staining of LSD1 and OTUD7B in normal tissue (*n* = 22) and the indicated breast carcinoma subtypes (luminal = 76, HER2^+^ = 49, and basal‐like = 130). Scale bars: 100 µm. G) Quantification of OTUD7B and LSD1 intensity in (F). ^***^ *P* < 0.001. *P* values were calculated by two‐tailed unpaired *t* test. H) TCGA DNA sequencing results showing that the *OTUD7B* gene is amplified at higher frequencies in basal‐like subtype (27%) compared to other subtypes (19%). I) Kaplan–Meier plots analysis of distant metastasis free survival rates (DMFS) in basal‐like breast cancer patient with high or low *OTUD7B* mRNA levels. Patient number at risk at different times of analysis is shown at the bottom of the plots. J) The working model of OTUD7B‐mediated LSD1 deubiquitination in coordinating LSD1 turnover, LSD1/CoREST corepressor complex assembly, and the subsequent impact on cell cycle and cancer metastasis.

LSD1 has been implicated in promoting cancer metastasis.^[^ ^24^, ^25^ ^]^ Using MDA‐MB231 cells, we found silencing OTUD7B or LSD1 markedly abrogated the migration and invasion of LM2 cells (Figure S7A, Supporting Information). By contrast, in LSD1^2KR^ cells, OTUD7B deficiency failed to reduce migration and invasion (Figure S7B, Supporting Information). These results were consistent with our RNA‐seq analysis revealed significant downregulation of genes known to promote metastasis upon OTUD7B or LSD1 depletion (Figure S7C, Supporting Information). Notably, OTUD7B or LSD1 knockdown showed no detectable effects on cell proliferation in these cells (Figure S7D,E, Supporting Information). A previous study reported a metastatic suppressor role of LSD1 in MDA‐MB‐231 cells.^[^ ^26^ ^]^ We therefore conducted rescue experiments by depleting LSD1 either in control cells or in LSD1^WT^ reconstituting cells. LSD1 reconstitution completely restored diminished migration and invasion potential triggered by LSD1 loss (Figure S7F, Supporting Information), excluding a possibly off‐target effects. We next performed fat pad injection, followed by quantitative bioluminescence imaging analyses. Similar to LSD1 depletion, OTUD7B knockdown significantly impaired lung metastasis of LM2 cells (**Figure** **7A**,B). Again, LSD1^2KR^ cells conferred profound resistance to loss in the metastatic potential when OTUD7B is deficient (Figure 7C).

We next explored the clinical implications of OTUD7B–LSD1 signaling. Increased *OTUD7B* transcript levels have been found across human cancers, particularly in breast cancers based on TCGA database (Figure S7G, Supporting Information). Immunohistochemical (IHC) staining detected profoundly elevated protein expression of both OTUD7B and LSD1 in 405 cases of primary breast cancer samples (Figure 7D; Figure S7H, Supporting Information), strongly correlating with their histological grades (Figure 7E). Moreover, a strong positive correlation between OTUD7B and LSD1 was also found in matched lymph node metastases (Figure S7I,J, Supporting Information). These observations prompted us to explore the enrichment of signaling pathways promoting metastasis based on OTUD7B/LSD1‐mediated gene expression signature. To this end, we utilized data from Human Cancer Metastasis Database (HCMDB)^[^ ^27^ ^]^ for gene set enrichment analysis (GSEA). Using OLco‐regulated gene sets revealed that loss of OTUD7B or LSD1 resulted in a significantly reduced expression of metastatic genes (Figure S7K, Supporting Information), providing potential mechanistic clues explaining suppressed metastatic ability due to impaired OTUD7B–LSD1 signaling. This result also indicates aberrantly high expression levels of these proteins might be of prognostic significance of tumor grade and lymph node metastasis in breast cancer patients.

IHC staining of OTUD7B and LSD1 in a cohort of 277 patients revealed a much stronger staining intensity of these two proteins in basal‐like subtype (*P* < 0.001) (Figure 7F,G). Further examination of published genomic data from METABRIC revealed significantly increased frequency of copy number gains of OTUD7B (57/209) in basal‐like subtypes as compared to other subtypes (273/1399) (Figure 7H).^[^ ^28^ ^]^ Consistent with this finding, basal‐like breast cancer patients with high *OTUD7B* expression had poor survival rates compared with the rest of the cohort, as indicated by distant metastasis‐free survival (DMFS) (log‐rank *P* = 0.013) was drastically decreased in *OTUD7B*‐high patients (Figure 7I), demonstrating aberrant OTUD7B expression might be a potentially useful biomarker in the prognosis prediction in basal‐like breast cancer patients.^[^ ^29^ ^]^ Collectively, these results highlight the clinical relevance of the dysregulated OTUD7B/LSD1 axis, which might serve as potential prognostic markers for poor survival outcome in breast cancer patients.

## 3 Discussion

Previous work suggests that LSD1 regulates distinct transcriptional outputs by interacting with different multicomponent complexes. Several post‐translational modifications of LSD1 have been reported to modulate its activity or stability.^[^ ^6^, ^7^, ^8^, ^30^ ^]^ Here, we unravel a pivotal role of ubiquitination in modulating the interaction of LSD1 with corepressor complex components. By removing K63‐linked polyubiquitination of LSD1 at K226/K277 residues, OTUD7B functions as a key player in determining the corepressor complex integrity. Meanwhile, OTUD7B loss facilitated LSD1 association with p62 in the nucleus, resulting in proteolytic degradation of LSD1. Thus, our findings have not only revealed a previously unknown p62‐dependent proteasomal pathway in regulating LSD1 stability, but also demonstrated the physiological importance of OTUD7B‐mediated deubiquitination of LSD1 in governing the switch of its binding partners, which ultimately impacts on the homeostasis and biological function of LSD1 (Figure 7J).

The intrinsic demethylase activity of LSD1 allows it to regulate cell fate decisions. Interestingly, LSD1 expression oscillates throughout the cell cycle, and OTUD7B protein levels coincide with LSD1 fluctuation kinetics. We further demonstrate this dynamic alteration of OTUD7B expression, leading to fluctuation of K63‐linked LSD1 polyubiquitination, accounts for cell cycle‐dependent LSD1 expression, highlighting an essential physiological role of OTUD7B in regulating LSD1 homeostasis. It is conceivable that to coordinate a wide variety of cellular processes, LSD1 demands a refined and complicated regulatory network consisting of antagonistic ubiquitination and deubiquitination pathways to maintain its dynamic homeostasis. Although the mode of action of E3(s)‐mediated LSD1 ubiquitination is far from clear, we speculate that several E3s might be involved in independent modulating LSD1 ubiquitination. Our findings that LSD1^2KR^ confers only partial yet significant resistance to CoREST deficiency‐induced LSD1 degradation, suggesting additional lysine residues might be targeted for CoREST‐mediated LSD1 polyubiquitination. One plausible explanation is that CoREST may compete for LSD1 binding with unknown E3(s), which catalyze K63‐linked ubiquitination of LSD1 in the nucleus. Meanwhile, USP7, USP22, and USP28 have been reported to deubiquitinate LSD1, leading to its stabilization under different experimental settings. These lines of evidence raise the possibility that LSD1 deubiquitination is catalyzed by different DUBs in a context‐specific manner. For instances, phosphorylation or methylation might be a prerequisite for LSD1 to gain access to certain DUBs. Nevertheless, our data suggest a crucial and unique role of OTUD7B among the known DUBs as a rate‐limiting factor determining LSD1 abundance throughout the cell cycle.

OTUD7B is known to regulate cell fate and cell proliferation via multiple signaling pathways.^[^ ^12^, ^13^ ^]^ Inhibition of LSD1 upon genetic approach or by small molecule compound has been shown to abrogate cell survival or induce G1 arrest in a cell type‐dependent manner.^[^ ^23^, ^31^ ^]^ We observed that loss of OTUD7B or LSD1 elicited drastic G1 arrest in breast cancer cells upon synchronization. Given their protein levels decline in G1 and peak in G2/M, this G1 arrest is rather unexpected. Cyclin D1 and CDK6, two of the OLco‐regulated targets, were largely diminished in their protein expression in unperturbed or synchronized cells when OTUD7B or LSD1 was silenced. However, in control cells upon synchronization, only CDK6 expression oscillates throughout the cell cycle, consistent with OTUD7B or LSD1 expression kinetics, whereas Cyclin D1 remains unchanged. These data highlight a dose‐limiting transcriptional regulation of genes involved in cell proliferation by OTUD7B–LSD1 axis, which might explain why this pathway confers a proliferative advantage in a context‐dependent manner.

In addition to LSD1, mitotic factors like Aurora A and Cyclin B have been identified as OTUD7B substrates.^[^ ^12^ ^]^ We noticed that knockdown LSD1 resulted in reduced Cyclin B levels in our system, reminiscent of changes seen in OTUD7B‐depleted cells. Intriguingly, in LSD1^2KR^ reconstituting cells, loss of OTUD7B failed to downregulate Cyclin B. These results raise the following possibilities. The impaired Cyclin B expression might be simply a direct consequence of a G1 arrest in cells depleted of OTUD7B or LSD1. Alternatively, OTUD7B‐dependent regulation of Cyclin B may rely on cofactors like LSD1. We certainly cannot formally rule out a possible context‐dependent co‐regulation of substrate proteins by OTUD7B with its cofactors.

Given expression levels of OTUD7B oscillate throughout the cell cycle, it is intriguing that this DUB is responsible for maintaining the homeostasis of substrate molecules at different cell cycle stages. Similar observations were obtained with crucial cell cycle regulators like anaphase‐promoting complex (APC). APC has been shown to degrade SCF component Skp2 in G1^[^ ^32^ ^]^ and target Cyclin B for degradation in G2,^[^ ^33^ ^]^ thereby controlling cell cycle progression at G1 and G2/M, respectively. Of note, APC levels drop in the G1 phase, kinase levels rise in M and early G1, ensuring the next phase of the cell cycle.^[^ ^34^ ^]^ It is plausible that the enzymatic activity of OTUD7B may also oscillate during the cell cycle progression. However, according to the deubiquitinating pattern of LSD1, the enzymatic activity of OTUD7B is expected to be low in G1 and high in G2/M, which just coincides with its protein oscillation pattern during the cell cycle. Dissecting upstream factors or possible enzymatic complex components regulating the deubiquitination activity of OTUD7B may shed light on this question.

In agreement with previous findings, where LSD1 has been found to facilitate metastasis across tumor types,^[^ ^35^ ^]^ OTUD7B depletion or LSD1 knockdown significantly suppresses the metastatic potential of breast cancer cells. A number of metastatic‐promoting genes were identified as OLco‐regulated targets, including angiogenic factors, chemokines, and epithelial‐mesenchymal transition factors like Snail. Therefore, the altered metastatic capacity might be attributed to changes of multiple pathways downstream of OTUD7B/LSD1 signaling. We noticed a previous study that showed LSD1 suppressed metastasis in vitro and in vivo using MDA‐MB‐231 cells,^[^ ^26^ ^]^ which is opposite to conclusions from our study and others.^[^ ^24^, ^36^ ^]^ This discrepancy might be attributed to different experimental settings utilized by independent groups.

Given a critical role of OTUD7B in modulating LSD1/CoREST complex integrity, we were curious to evaluate the influence of OTUD7B on LSD1‐dependent transcriptome at the global scale. A remarkable loss of global LSD1 binding and a very significant gain of global H3K4me2 were found upon OTUD7B depletion. Gene transcription profiling analysis further revealed a critical role of OTUD7B in modulating LSD1‐dependent transcriptome. A substantial population of genes is found to be co‐regulated by OTUD7B and LSD1, including genes essential for modulating metastatic capacity and genes required for G1/S transition. Given the primary action of K63‐linked polyubiquitination of LSD1 is to hamper LSD1/CoREST complex formation, this event may not cause any genome‐wide discrimination between transactivation and transrepression properties of LSD1. The alterations in both activating and repressive histone marks on OLco‐regulated genes support this notion. We certainly cannot formally exclude the possibility that, under specified conditions, OTUD7B may affect the association of LSD1 with interacting partners that determine the substrate specificity of LSD1. Nevertheless, our data highlight a prominent role of OTUD7B in modulating formation of functional LSD1/CoREST complex in the process of gene transcription.

LSD1 is generally believed to function as an oncoprotein. Pharmacological targeting of LSD1/CoREST/HDACs complex has showed very promising antitumor effect in human cancers.^[^ ^31^ ^]^ Of note, in addition to modulate its histone substrates, LSD1 has been shown to repress tumor suppressors like p53 and FBXW7,^[^ ^37^ ^]^ as well as to activate oncogenic signaling like androgen‐receptor‐dependent transcription^[^ ^38^ ^]^ via distinct mechanisms. It will be interesting to explore a possible interplay between OTUD7B and these signaling pathways connected by LSD1, which will broaden our viewpoint on the pathophysiological functions of OTUD7B. Meanwhile, emerging evidence suggest OTUD7B possesses oncogenic properties by modulating deubiquitination of several known oncoproteins.^[^ ^12^, ^13^, ^14^ ^]^ Given a critical role of OTUD7B in regulating LSD1/CoREST repressor complex, OTUD7B may serve as an attractive target for therapeutic intervention. Furthermore, several lines of evidence, including aberrant expression of OTUD7B, and its tight correlation with high tumor grades and shorter metastasis free survival, indicate OTUD7B as a potential marker for predicting adverse prognosis in breast cancer patients.

## 4 Experimental Section

### Cell Culture and Transfection

LM2 was a kind gift from Guohong Hu (University of Chinese Academy of Sciences) and U251 was gifted from Yin Chen (Xiamen University). Other cell lines were obtained from ATCC. HEK293T, MDA‐MB‐231, A549, HeLa, HT1080, RKO, SW480, SW620, MCF‐7, LM2, and U251 cells were cultured in DMEM (Gibco) supplemented with 10% FBS (Gemini); T‐47D, BT549, H460, H1299, DLD1, NCI‐H358, and 786‐O cells were cultured in RPMI‐1640 (Gibco) supplemented with 10% FBS; HCT116, U2OS, HT29, and SKOV3 cells were culture in McCoy's 5A (Sigma) supplemented with 10% FBS. All cells were maintained at 37 °C in a saturated humidity atmosphere containing 95% air and 5% CO_2_. For HEK293T cells, transfections were performed using the calcium‐phosphate precipitation method.

### Cloning Procedures

Expression vectors HA‐DUBs, HA‐CoREST, and HA‐p62 were generated by subcloning the corresponding cDNAs into the pCDNA3.1‐HA expressing vector via BamHI and XhoI sites. Myc‐OTUD7B and Myc‐LSD1 were generated by subcloning the corresponding cDNAs into the pCDNA3.1‐Myc expressing vector via BamHI and XhoI sites. Flag‐LSD1, HA‐OTUD7B, and HA‐p62 were generated by subcloning the related pCDNA3.1 constructs into the pLV‐EGFP expressing vector via BamHI and XhoI sites. GST‐OTUD7B and GST‐LSD1 were generated by subcloning the corresponding cDNAs into the pGEX4T1 expressing vector via BamHI and XhoI sites. His‐LSD1 and His‐OTUD7B were generated by subcloning the corresponding cDNAs into the pET21b expressing vector via BamHI and XhoI sites. GST‐LSD‐SWIRM (aa 1–260), GST‐LSD1‐AOD‐1+TOWER (aa 261–520), and GST‐LSD1‐AOD‐2 (aa 521–852) were generated by subcloning the corresponding cDNAs into the pGEX4T1 expressing vector via BamHI and XhoI sites. The Flag‐LSD1 (K226R/K280R), HA‐OTUD7B (C194A/H358R), HA‐p62 shRNA‐resistant constructs, HA‐OTUD7B sgRNA‐resistant construct, Flag‐LSD1 shRNA‐resistant construct, and Myc‐OTUD7B‐ΔOTU (deleting amino acid(aa) 189–359), Myc‐OTUD7B‐ΔZNF (deleting aa 810–843), Myc‐OTUD7B‐ΔUBA (deleting aa 1–40), and Myc‐OTUD7B‐ΔZNF+UBA (deleting aa 1–40 and 810–843) mutants were constructed using the Site‐Directed Mutagenesis Kit (Stratagene) following the manufacturer's instruction. HA‐tagged Ub constructs were the kind gifts from Dr. Zhijian James Chen (University of Texas Southwestern Medical Center, Dallas, Texas, USA). And, Myc‐Ub constructs were kindly provided by Dr. Zongping Xia (The First Affiliated Hospital of Zhengzhou University, Zhengzhou, China).

### DUB Screening

HA‐ or Flag‐tagged deubiquitinase plasmids were introduced into HEK‐293T cells by calcium‐phosphate precipitation method. 48 h later, cells were lysed by lP buffer containing 150 × 10^−3^ m NaCl, 50 × 10^−3^ m Tris‐HCl pH = 7.4, 40 × 10^−3^ m *β*‐glycerophosphate, 1 × 10^−3^ m Na_4_OV_3_, 10 × 10^−3^ m NaF, and 2 × 10^−3^ m ethylene diamine tetraacetic acid (EDTA) supplemented with 1 × 10^−3^ m phenylmethanesulfonyl fluoride (PMSF) and protease inhibitor (Roche). Cell lysates were incubated with anti‐HA or anti‐Flag antibody‐conjugated agarose beads for 4 h and then washed five times with immunoprecipitation (IP) buffer, followed by western blotting analysis.

### Lentiviral Infection

Lentiviral‐based vector pLV‐H1‐EF1*α* (Biosettia) and Lenti‐CRISPR were used for RNA interference experiment. Lentivirus was produced by co‐transfecting HEK293T cells with the shRNA. Viral supernatant was harvested at 48 h post‐transfection, passed through a 0.45 µm filter, and used to infect the target cells at 80% confluence in the presence of protamine sulfate (8 µg mL^−1^). shRNA or sgRNA used in this paper is listed in Table S4 in the Supporting Information.

### Immunoprecipitation and Immunoblotting (IB)

The immunoprecipitation detailed procedure was performed as previously described.^[^ ^39^ ^]^ In brief, cells were lysed by lP buffer containing 150 × 10^−3^ m NaCl, 50 × 10^−3^ m Tris‐HCl pH = 7.4, 40 × 10^−3^ m *β*‐glycerophosphate, 1 × 10^−3^ m Na_4_OV_3_, 10 × 10^−3^ m NaF, and 2 × 10^−3^ m EDTA supplemented with 1 × 10^−3^ m PMSF and protease inhibitor (Rche). Cell lysates were incubated with antibodies overnight at 4 °C. Protein A/G beads were added and 2 h later washed five times with IP buffer and then subjected to western blotting analysis.

For immunoblotting analysis, cultured cells were lysed by RIPA lysis buffer (150 × 10^−3^ m NaCl, 50 × 10^−3^ m Tris‐HCl pH = 7.4, 1% Triton X‐100, 1% sodium deoxycholate, 0.1% SDS). Protein concentration of each sample was determined by using the BCA kit (Pierce) as manufacturer's instructions. Equal amounts of protein extracts were separated by electrophoresis on appropriate Tris‐Glycine gel, and then transferred to a nitrocellulose membrane (Millipore). The membrane was probed with different primary antibodies, followed by secondary antibodies conjugated to horseradish peroxidase. Quantitative densitometry analysis was performed with image analysis software (Quantity One, BioRad).

For ubiquitination assay, cells were treated with 100 × 10^−9^ m BTZ for 5 h or 25 × 10^−6^ m MG132 for 8 h, and then collected and lysed by modified RIPA lysis buffer (50 × 10^−3^ m Tris‐HCl pH = 8.0, 0.1% SDS, 1% sodium deoxy acid, 1% Triton X‐100, 150 × 10^−3^ m NaCl, 1 × 10^−3^ m Na_4_VO_3_, 10 × 10^−3^ m NaF, and 1 × 10^−3^ m EDTA) supplemented with 5 × 10^−3^ m NEMI and 1 × 10^−3^ m PMSF. Cell lysates were denatured by boiling for 5 min in the presence of 1% SDS, and then cooled down on ice for 3 min, followed by centrifugation at room temperature for 5 min. The lysates were subject to immunoprecipitation with indicated antibodies and analyzed by IB analysis. Antibodies used are listed in Table S5 in the Supporting Information.

### Immunofluorescence

Cells were seeded on 6‐well plate with coverslips and followed by synchronized in the G2/M phase by sequential thymidine (2 × 10^−3^ m) and nocodazole (200 ng mL^−1^) block. The culture medium was removed and coverslips were carefully washed three times with PBS. Then, cells were fixed by 4% paraformaldehyde for 5 min at room temperature and subsequently washed twice with PBS and twice with washing buffer. Cells were then permeabilized with 0.5% Triton X‐100 for 5 min, blocked in PBS plus 1% BSA, and subsequently incubated with primary antibodies against OTUD7B (1:300, Proteintech, Cat# 16605‐1‐AP) and LSD1 (1: 400, Cell signaling, Cat# 4218) at 4 °C overnight. After washing three times with PBS, the cells were incubated with Alexa Fluor 488 goat antirabbit (Life Technologies) and Alexa Fluor 568 donkey antimouse (Life Technologies) at room temperature for 1 h in the dark. After extensive washing with ice‐cold PBS, the nuclei were counterstained with DAPI for 10 min. Then, specimens were mounted in 70% glycerol and sealed with nail polish. The fluorescence images were taken by structured illumination microscopy (SIM) (GE OMX V4).

### GST Pull‐Down Assay

The GST pull‐down assay was performed as before.^[^ ^39^ ^]^ Briefly, indicated GST fusion constructs were expressed in BL21, and crude bacterial lysates were prepared by sonication in cold NETN buffer (50 × 10^−3^ m Tris‐HCl pH = 8.0, 120 × 10^−3^ m NaCl, and 1 × 10^−3^ m EDTA, pH = 8.0, 0.5% NP‐40) in the presence of the protease inhibitor mixture. Then, GST fusion proteins were purified by glutathione‐sepharose beads. In GST pull‐down assays, about 10 µg of the appropriate GST fusion proteins were mixed with cell lysates or purified proteins. The binding reaction was mixed at 4 °C for 2 h. The beads were washed five times with NETN resuspended in 15 µL of 2× SDS‐PAGE loading buffer, and resolved on the appropriate Tris‐Glycine gel. Protein bands were detected with specific antibodies by western blotting.

### In Vitro Deubiquitination Assay

HEK293T cells transfected with Myc‐tagged LSD1 and HA‐tagged Ubiquitin were treated with 100 × 10^−9^ m BTZ for 5 h. LSD1 immunoprecipitates containing ubiquitinated LSD1 was purified from the cell lysates using Myc‐beads and washed extensively using RIPA washing buffer (50 × 10^−3^ m Tris‐HCl, pH = 8.0, 140 × 10^−3^ m NaCl, and 1 × 10^−3^ m EDTA, 0.1% Triton X‐100). The proteins were then eluted with Myc‐peptides (Sigma) in RIPA washing buffer. The recombinant GST‐tagged OTUD7B protein was expressed in BL21 cells and purified using GST beads. For the in vitro deubiquitination assay reaction, the ubiquitinated LSD1 protein was incubated with purified GST‐tagged OTUD7B protein in a deubiquitination buffer (50 × 10^−3^ m Tris‐HCl pH = 7.5, 150 × 10^−3^ m NaCl, 2 × 10^−3^ m EDTA pH = 8.0, and 2 × 10^−3^ m dithiothreitol (DTT)) at 37 °C overnight, followed by western blotting analysis.

### In Vitro Demethylase Assay

The in vitro demethylase assay was performed as described before.^[^ ^40^ ^]^ In brief, H3K4me1 peptide was incubated with the indicated purified recombinant GST‐LSD1 constructs in MT buffer (80 × 10^−3^ m Tris‐HCl pH = 8.0, 200 × 10^−3^ m NaCl, 4 × 10^−3^ m EDTA, 12 × 10^−3^ m MgCl_2_, 0.4 µg mL^−1^ BSA, and 4 × 10^−3^ m DTT) for 1 h at 37 °C, followed by mass spectrometry analysis.

### SILAC, Affinity Purification, in Solution Digestion, and LC‐MC/MS Analysis

MDA‐MB‐231 cells expressing control shRNA or CoREST shRNA were cultured in SILAC DMEM supplemented l‐lysine/arginine and l‐lysine/arginine‐U‐13C6 with 10% dialyzed FBS, l‐glutamine, and penicillin/streptomycin for 2 weeks followed by infection with lentiviral vector expressing Flag‐tagged LSD1. After infection for 96 h, cells were treated with 100 × 10^−9^ m BTZ for 5 h and the nucleus lysates were extracted using buffer containing 20 × 10^−3^ m HEPES (pH = 7.6), 1.5 × 10^−3^ m MgCl_2_, 420 × 10^−3^ m NaCl, 0.5 × 10^−3^ m DTT, 0.5 × 10^−3^ m PMSF, 25% glycerol, and 1% NP‐40 after removing cytoplasmic proteins by cell lysis buffer (50 × 10^−3^ m Tris‐HCl pH = 8.0, 0.5% NP40 and 50 × 10^−3^ m NaCl). Lysates were pooled and then immunoprecipitated by Flag‐M2 beads. The Flag‐immunoprecipitates were eluted by 3× Flag peptides and then subjected to M/S analysis as described before.

### Flow Cytometry‐Based Cell Cycle Analysis

For G2/M synchronization, cells were first treated with 2 × 10^−3^ m thymidine for 16 h, washed two times with PBS, and released into complete media containing nocodazole (200 ng mL^−1^) for 14 h.

For cell cycle analysis, cells were collected and washed with PBS, fixed with 70% ethanol, and resuspended in PBS containing PI (20 µg mL^−1^) and RNase A (200 µg mL^−1^). Analyses were performed using flow cytometer BD LSRFortessa (BD biosciences) and FlowJo software (Treestar, Ashland, USA).

### Quantitative Real‐Time PCR

Total RNA was isolated from samples with Trizol following the manufacturer's instructions (Invitrogen). cDNA was prepared with Primescript Reverse Transcriptase Kit (Invitrogen). Quantitative PCR was performed using the StepOne‐plus real‐time PCR system (Applied Biosystems Inc., Foster City, CA) that measures real‐time SYBR green fluorescence and then calculated by means of the comparative Ct method with the expression of GAPDH as an internal control. The primers used for the real‐time PCR experiments are listed in Table S6 in the Supporting Information.

### Chromatin Immunoprecipitation Assay and ChIP‐Seq

ChIP assays were performed as previously described.^[^ ^39^, ^41^ ^]^ Briefly, cells were fixed with 1% formaldehyde (Sigma) for 10 min at room temperature (RT) (for H3Kme2 and H3K9me2 ChIP), or fixed with DSG (2 × 10^−3^ m) (Proteochem) for 45 min at RT (for LSD1 ChIP), and washed twice with PBS. Fixation was stopped by adding glycine (0.125 m) and incubated for 5 min at RT, followed by washing with PBS twice. Cells were then lysed using cell lysis buffer (50 × 10^−3^ m Tris‐HCl pH = 8.0, 0.5% NP40 and 50 × 10^−3^ m NaCl) to remove the cytosol proteins. The pellet was resuspended in 1% SDS lysis buffer (for H3K4me2 or H3K9me2 ChIP) or nuclear lysis buffer (1% Triton X‐100, 2 × 10^−3^ m EDTA pH = 8.0, 400 × 10^−3^ m NaCl, 20 × 10^−3^ m Tris‐HCl pH = 7.8, and 0.1% SDS) (for LSD1 ChIP). Chromatin DNA was sheared to 300–500 bp average in size through sonication. The lysates were immunoprecipitated with control IgG or the indicated specific antibodies overnight at 4 °C, followed by incubation with protein G magnetic beads (Invitrogen) for 4 h. After washing and elution, the protein–DNA complex was reversed by heating overnight at 65 °C. Immunoprecipitated DNA was purified by using QIAquick spin columns (Qiagen) and subjected to high‐throughput sequencing. Relative ChIP enrichment was confirmed via qPCR. The primers used for the ChIP‐qPCR experiments are listed in Tables S7–S9 in the Supporting Information.

### Analysis of DMFS of Breast Cancer Patients

Kaplan–Meier survival curves were generated using the Kaplan–Meier Plotter website for Breast cancer (Version 2020, http://kmplot.com) and statistical significance was determined by the log‐rank test.

### Immunohistochemistry

Formalin‐fixed paraffin‐embedded 5 µm tissue sections were deparaffinized in dimethylbenzene and rehydrated through a graded series of alcohols. After antigen retrieval was performed, all sections were blocked at room temperature in avidin/biotin blocking buffer (Vector Laboratories) and then 3% BSA for 30 min. Staining with antibodies was conducted overnight at 4 °C. Sections were rinsed twice in PBS, and protein staining was performed using a diaminobenzidine (DAB) substrate kit (Maxim). Samples were counterstained with hematoxylin (BOSTER). Immunohistochemistry images were obtained using an upright microscope (Leica DM4B).

### Transwell Assay

For in vitro migration assay, an 8 µm pore size Boyden chamber (Corning Costar) was used. Cells (100 µL, 1 × 10^5^) in 0.5% serum‐containing DMEM were plated in the upper chamber, and 600 µL 10% FBS was added to DMEM in the lower chamber as a chemoattractant. For invasion assay, an 8 µm pore size BD Matrigel Invasion Chamber was used. After 3 h for migration assay and 6 h for invasion assay, cells on the upper side of the filter were removed and cells that remained adherent to the underside of membranes were fixed in methanol, followed by staining with crystal violet. The number of migrated cells was counted using a microscope. Five contiguous fields of each sample were examined using a 20× objective to obtain a representative number of cells that migrated/invaded across the membrane.

### Cell Proliferation Assay

Cells stably infected with the indicated lentiviruses were plated in 6‐well plates in triplicates. The number of viable cells per well at each time point was measured using Z2 coulter particle count and size analyzer (Beckman Coulter, Brea, CA, USA) according to the manufacturer's instructions.

### Animal Studies

Female nude mice (BALB/C, 15–20 g, 4–6 weeks old) were obtained from Shanghai SLAC Laboratory Animal Technology, China, and maintained under pathogen‐free conditions. For metastasis formation, LM2 cells were harvested, washed twice in PBS, counted, and then resuspended in a 1:1 solution of PBS and Matrigel Matrix (Corning). Mice were anesthetized, a small incision was made to reveal the no. 4 mammary fat pad, and luciferase‐labeled 3 × 10^6^ parental or LSD1 reconstituting LM2 cells were injected directly into the mammary fat pad. When tumors became palpable, tumor volume was assessed by caliper measurements using the following formula: *π* (width × length)/6 (mm^3^). The tumors were extracted from mammary glands when they reached 300 mm^3^. Seven days after tumor removal, mice were monitored by bioluminescent imaging for the development of metastases. For bioluminescent imaging and analysis, mice were anesthetized and injected with 1.5 mg of d‐luciferin (15 mg mL^−1^ in PBS). Imaging was completed between 2 and 5 min after injection with a Xenogen IVIS Lumina system coupled to Living Image acquisition and analysis software (Xenogen). Images were analyzed with Living Image software ver.3.0. Bioluminescent flux (photons s^−1^ cm^−2^ steradian^−1^) was determined for the mouse in a prone position.

### Computational Analysis of ChIP‐Seq Data

The sequencing reads were aligned against human reference genome hg19 using Bowtie2^[^ ^42^ ^]^ (http://bowtie‐bio.sourceforge.net/bowtie2/index.shtml) with default parameters. Only sequences mapped uniquely to the genome with <2 mismatches were used for downstream analysis.

Clonal amplification was circumvented by allowing maximal one tag for each unique genomic position. The identification of ChIP‐seq peaks was performed using HOMER^[^ ^43^ ^]^ (http://homer.ucsd.edu/homer/) with default parameter. Genomic distribution was done by using the default parameters from HOMER with minor modifications, in which promoter peaks were defined as those with peak center falling between 2000 bp downstream and 5000 bp upstream of transcript start sites (TSS).

To define OTUD7B‐regulated LSD1 binding sites, only when FC of ChIP‐seq tag density of a peak in OTUD7B silencing versus control was larger than 2, that peak was classified into OTUD7B affected LSD1 binding sites (*P* < 0.0001). Tag density for histograms (25 bp per bin) and box plots were generated by using HOMER. Box plots were then generated by R software (https://www.r‐project.org/) and significance was determined using Student's *t* test.

### Computational Analysis of RNA‐Seq Data

Sequencing reads were aligned to the human reference genome (hg19) by STAR aligner.^[^ ^44^ ^]^ Cufflinks was used to calculate the expression of RefSeq annotated genes with the option ‐M (reads aligned to repetitive regions were masked) and ‐u (multiple aligned read are corrected using “rescue method”).^[^ ^45^ ^]^ Coding genes with fragments per kilobase per million mapped reads (FPKM) larger than 0.5 in control sample, OTUD7B‐, or LSD1‐depleted sample were included in the analysis. For differentially expressed genes, fold change larger than 1.5 was applied. Heat map was generated and visualized by deepTools.^[^ ^46^ ^]^ Significance was determined by student's *t* test.

### GSEA

GSEA was conducted by java GSEA Desktop Application^[^ ^47^ ^]^ with default parameters and considered gene set containing reported prometastatic genes associated with breast cancer selected from HCMDB. Gene list is shown in Table S10 in the Supporting Information.

### Statistical Analysis

Results were reported as mean ± SEM of 3 or more independent experiments. Student's *t* test was used to determine significance between groups. All *P* values reported were 2 sided unless otherwise noted. Fisher's exact test was used to determine significance of correlation. *P* < 0.05 was considered statistically significant. Statistical analyses were performed with GraphPad Prism 8 software

### Study Approval

Prior to obtaining patient samples, requisite approval from the Medical Ethics Committee of the First Affiliated Hospital of Xiamen University and written informed consent from the patients were obtained. The mouse experiments were approved by the Institutional Animal Care and Use Committee of Xiamen University (XMULAC 20170331).

## Conflict of Interest

The authors declare no conflict of interest.

## Author Contributions

Z.G. and A.L. contributed equally to this work. This project was conceived and supervised by H.Y. Some experiments were supervised by R.H., W.L., D.Z., and L.C. The experiments were designed by H.Y. and Z.G. Most of the experiments were performed by Z.G. and A.L. RNA‐seq and ChIP‐seq were analyzed by J.D. and W.L. Q.L., L.Z., Y.L., Z.M., F.C., and J.H. performed a specific subset of the experiments and analysis. The breast cancer sample collection and immunohistochemical analysis were contributed by J.Y. The manuscript was prepared by Z.G., R.H., and H.Y.

## Supporting information

Supporting Information

## Data Availability

The data that support the findings of this study are available from the corresponding author upon reasonable request.
