# Supplementary material for: OTUD7B Deubiquitinates LSD1 to Govern Its Binding Partner Specificity, Homeostasis, and Breast Cancer Metastasis
Source: Adv Sci (Weinh). 2021 May 29;8(15):2004504. doi: 10.1002/advs.202004504 (PMC8336515; doi:10.1002/advs.202004504)
Supplement: Supplementary file 1 — Supporting Information [file ADVS-8-2004504-s001.pdf]

© 2021 Wiley-VCH GmbH

ADVANCED SCIENCE
Open Access

Supporting Information

for *Adv. Sci.,* DOI: 10.1002/advs.202004504

OTUD7B Deubiquitinates LSD1 to Govern Its Binding Partner Specificity, Homeostasis and Breast Cancer Metastasis

*Zhicheng Gong, Aicun Li Jiancheng Ding, Qing Li, Lei Zhang, Yuanpei Li, Zhe Meng, Fei Chen, Jialiang Huang, Dawang Zhou, Ronggui Hu, Jing Ye, Wen Liu, and Han You**

Supporting Information

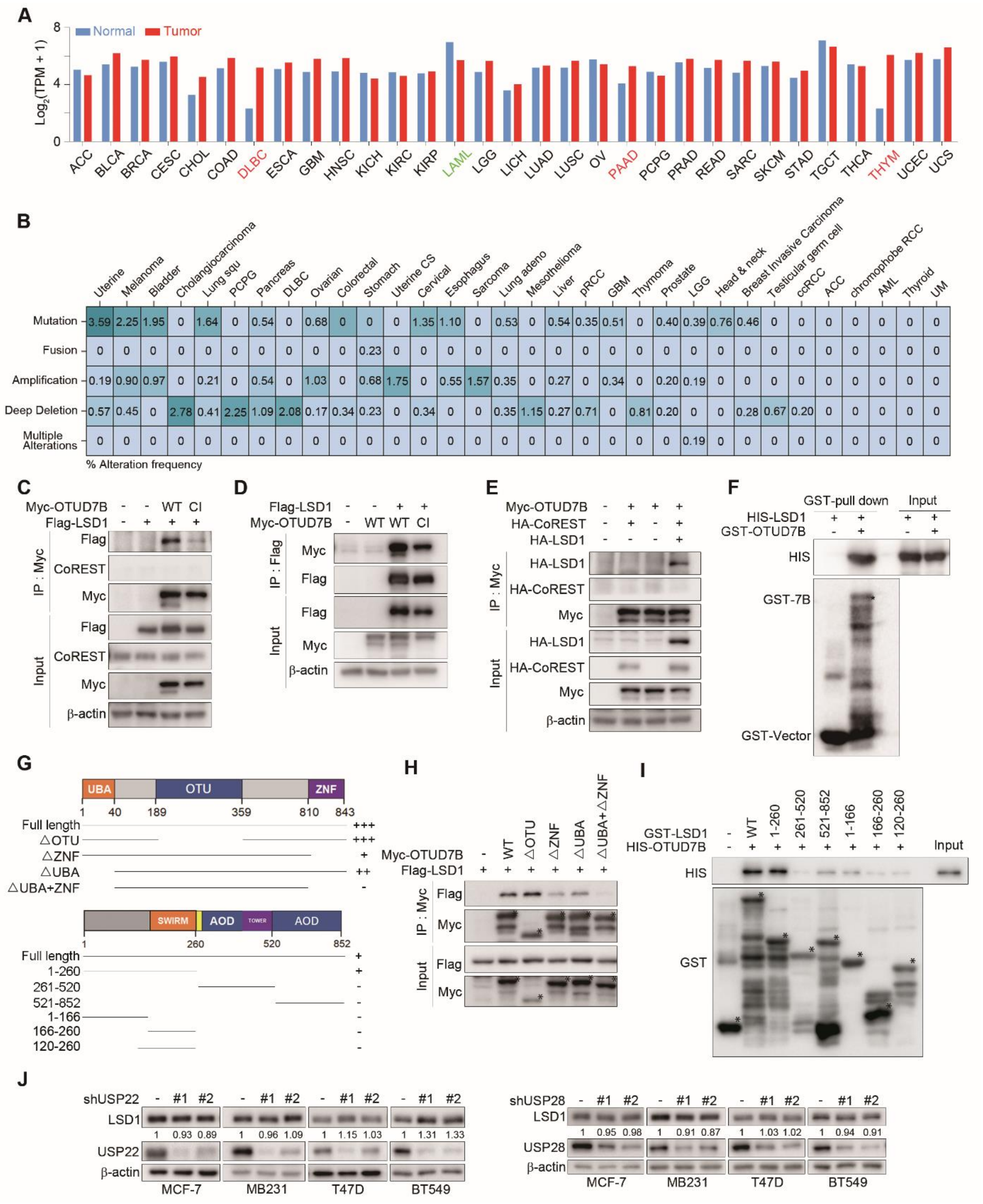

Figure S1. OTUD7B binds LSD1 and regulates its stability. A) Histogram shows the median mRNA expression profile of *LSD1* across the indicated tumor samples (red) and paired tissues (blue) using TCGA database. The cancer types with *LSD1* expression levels greater than 2-fold are highlighted in red. B) TCGA DNA sequencing results showing *LSD1* genomic alteration frequencies in the indicated tumor samples (n = 10953). (C-E) HEK293T cells were

transfected with the indicated plasmids, followed by IP and IB analysis as indicated. F) Purified His-LSD1 was incubated with GST or GST-OTUD7B coupled to GSH-Sepharose. Proteins retained on sepharose were analyzed by western blotting with the indicated antibodies. G) Schematic diagrams of deletion mutants of OTUD7B (top panel) and LSD1 (bottom panel). H) HEK293T cells were co-transfected with Flag-LSD1 and the indicated OTUD7B constructs, followed by IP and IB analysis as indicated. I) Purified His-OTUD7B was incubated with the indicated GST proteins coupled to GSH-sepharose. Proteins retained on sepharose were analyzed by western blotting. J) IB analysis of breast cancer cell lines infected with the indicated lentiviral shRNAs constructs.

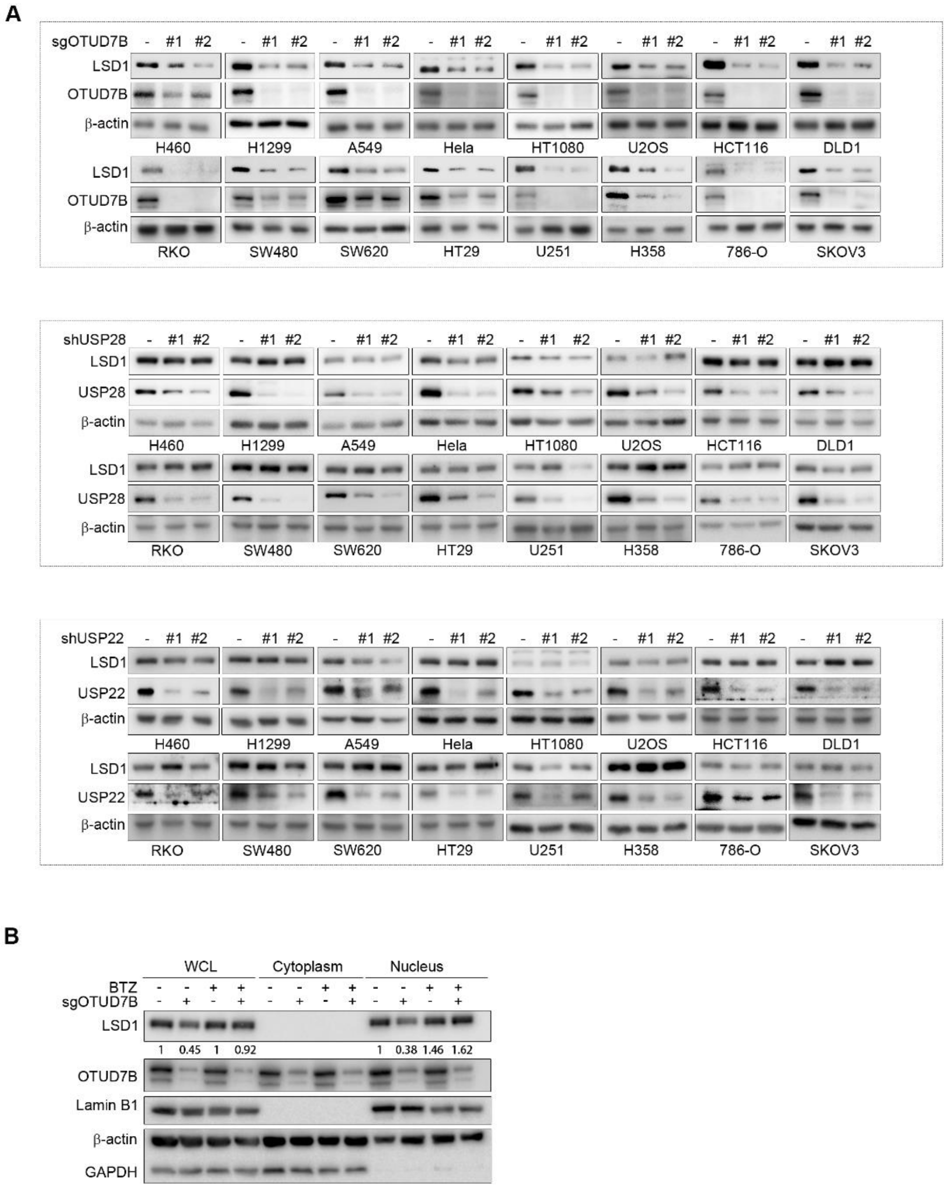

Figure S2. OTUD7B binds LSD1 and regulates its stability. A) IB analysis of WCL derived from 16 different cancer cell cells expressing the indicated lentiviral constructs. B) MDA-MB-231 cells expressing OTUD7B sgRNA were treated with BTZ for 5 hr, then lysed and subjected to IB analysis of LSD1 and OTUD7B in nuclear and cytoplasmic fractions using Lamin B1 (nuclear), GAPDH (cytoplasm) and β-actin (WCL) as controls.

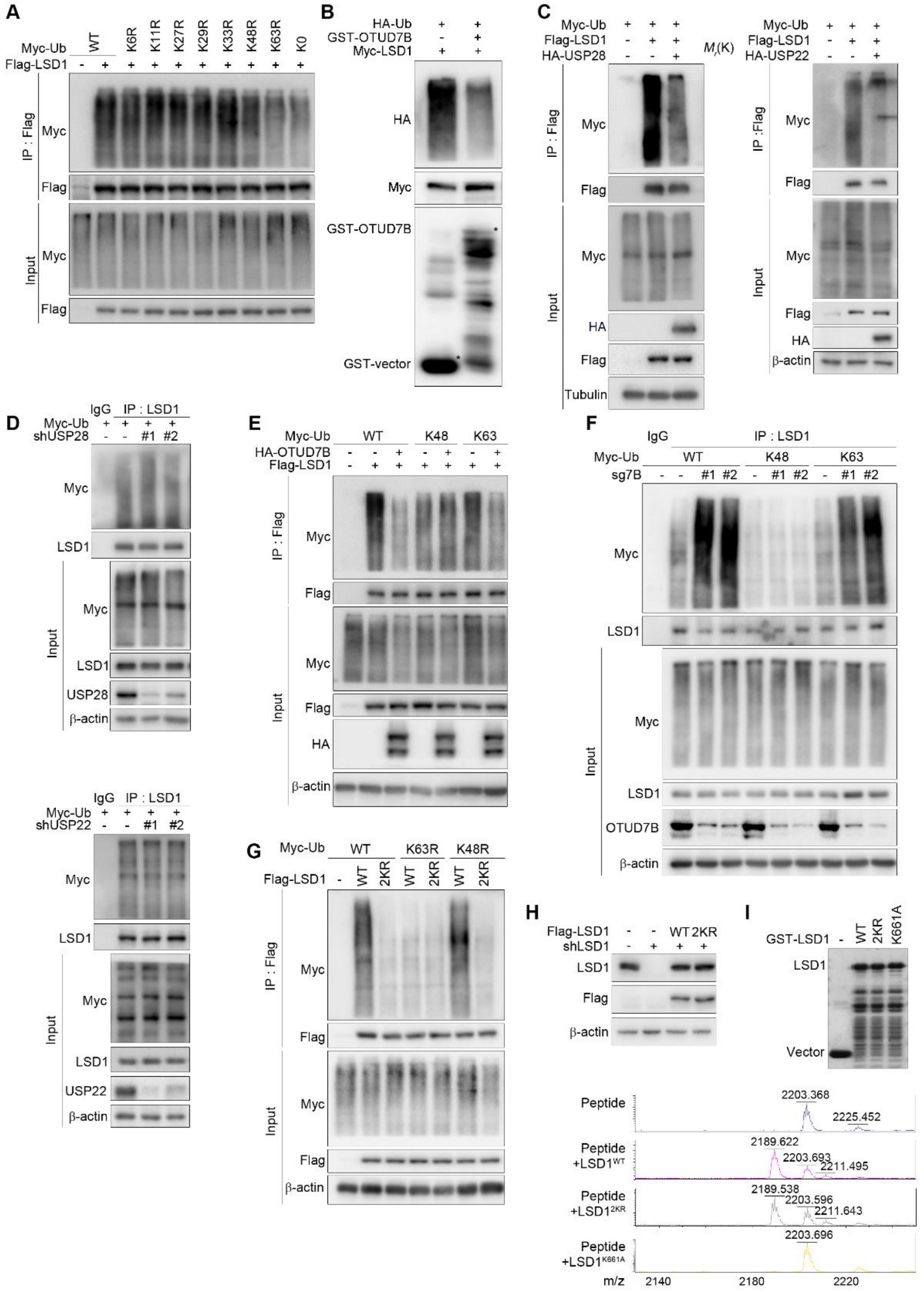

Figure S3. OTUD7B is a bona fide LSD1 deubiquitinase. A) HEK293T cells were co-transfected with Flag-LSD1 and the indicated Ubiquitin constructs, collected for Flag-IP, followed by IB analysis with the indicated antibodies. B) Ubiquitinated LSD1 obtained from HEK293T cells transfected with Myc-LSD1 and HA-ubiquitin followed by Myc-immunoprecipitation was incubated with GST or GST-OTUD7B. Proteins retained were analyzed by western blot with indicated antibodies. C) HEK-293T cells were co-transfected

with the indicated plasmids, collected for Flag-IP, followed by IB analysis with the indicated antibodies. D) HEK-293T cells infected with lentiviruses encoding the indicated shRNAs were transfected with Myc-Ubiquitin, treated with MG132 (25 μм) for 8 hr. Cell lysates were collected for IP with antibody against LSD1 and IB analysis as indicated. E) HEK293T cells were co-transfected with the indicated constructs, collected for Flag-IP, followed by IB analysis with the indicated antibodies. F) HEK293T cells stably expressing control or OTUD7B sgRNA were transfected with indicated Ub constructs and collected for LSD1-IP, followed by western blotting analysis with indicated antibodies. G) HEK293T cells were co-transfected with the indicated constructs, collected for Flag-IP, followed by IB analysis with the indicated antibodies. H) Western blotting analysis of LSD1 protein levels in $LSD1^{WT}$ and $LSD1^{2KR}$ reconstituting MDA-MB-231 cells. I) Coomassie Brilliant Blue (CBB) staining showing expression levels of the indicated GST-LSD1 recombinant proteins (top panel). In vitro demethylation assay was performed by incubating H3K4me1 peptides with the indicated GST-LSD1 constructs and analyzed by mass spectrometry (bottom panel).

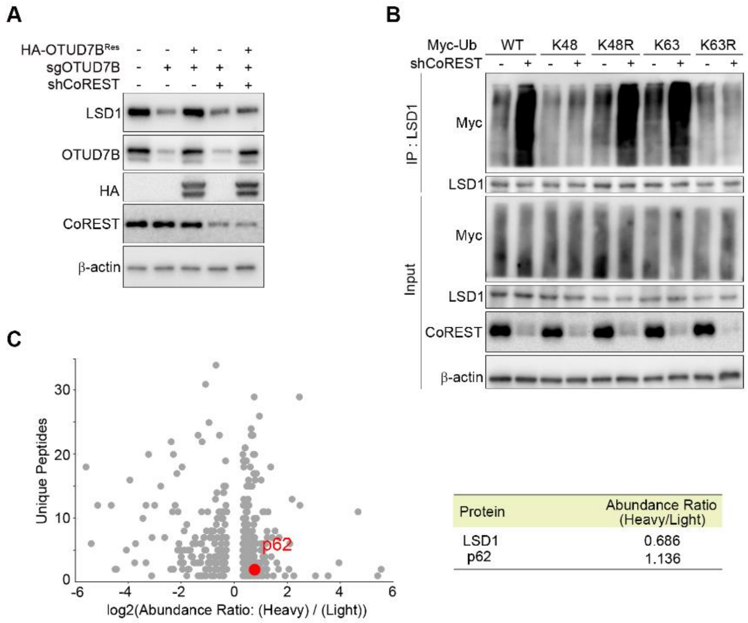

Figure S4. OTUD7B-mediated removal of K63-linked poly-Ub chains on LSD1 determines LSD1/ CoREST/HDACs integrity. A) MDA-MB-231 infected with lentiviruses encoding the indicated constructs, followed by IB analysis with the indicated antibodies. B) HEK293T cells expressing control or CoREST shRNA were transfected with the indicated Myc-tagged ubiquitin constructs, treated with BTZ for 5 hr, collected for IP with LSD1 antibody and IB analysis with the indicated antibodies. C) MDA-MB-231 cells labelled by SILAC were infected with lentiviruses encoding control or CoREST shRNA, treated with BTZ for 5 hr. Cell lysates were collected for IP with LSD1 specific antibody, followed by mass spectrometry analysis. Scatter plot represents the indicated LSD1-interacting proteins (p62 highlighted by red) (left panel); abundance ratio of p62 upon CoREST-depleted versus control condition (right panel).

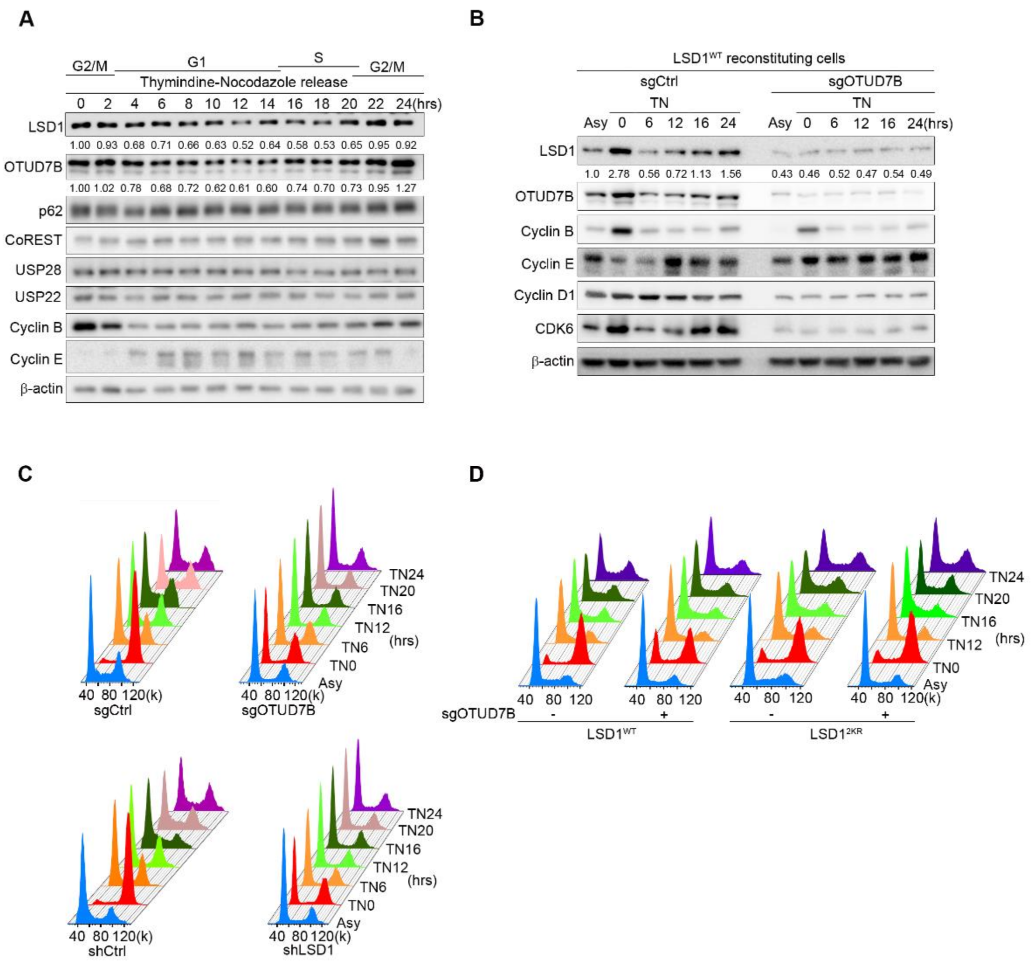

Figure S5. OTUD7B-dependent regulation of LSD1 fluctuation is crucial for cell cycle progression. A) MDA-MB-231 cells were synchronized by thymidine/nocodazole treatment, released and harvested at the indicated times, followed by western blotting analysis as indicated. B) LSD1^{WT} reconstituting MDA-MB-231 cells stably expressing control or OTUD7B sgRNA were synchronized by thymidine/nocodazole treatment, released and harvested at the indicated times, followed by western blotting analysis as indicated. (C-D) Parental (C) or LSD1 reconstituting D) MDA-MB-231 cells stably expressing the indicated shRNAs or sgRNAs were synchronized by thymidine/nocodazole treatment, released and harvested at the indicated times, followed by FACS analysis.

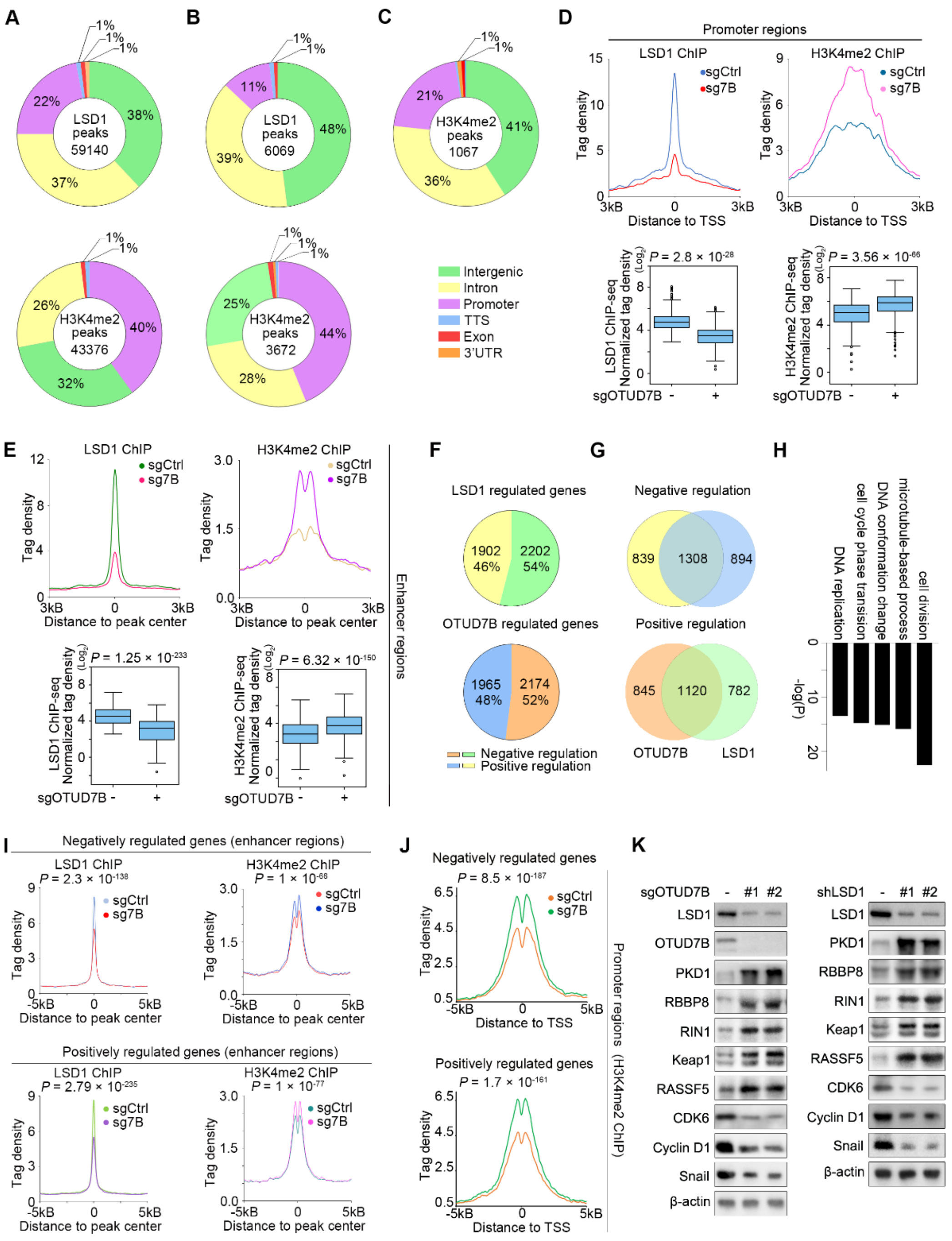

Figure S6. OTUD7B regulates gene transcription in a LSD1-dependent manner. A) Pie charts showing genomic distribution of LSD1-bound peaks (59,140) (top panel) and H3K4me2 peaks (43,376) (bottom panel). B) Pie charts showing genome-wide distribution of reduced LSD1-bound peaks (n = 6069, FC > 2) (top panel) and elevated H3K4me2 peaks (n = 3672, FC > 2) (bottom panel) upon OTUD7B loss. C) Pie chart showing genomic distribution of H3K4me2 peaks gained upon OTUD7B depletion, centered on reduced LSD1 peaks (± 3 kB) (n = 1067, FC > 1.5). (D-E) Fold enrichment of normalized LSD1 and H3K4me2 ChIP-seq reads at promoter regions (n = 223) (D) and enhancer regions (n = 844) (E) in MDA-MB-231

cells depleted of OTUD7B. Top panels show tag density distribution, and bottom panels show box plot. *P* values were calculated by student's t test. F) Pie charts showing differentially expressed genes detected by RNA-seq in LSD1-silenced or OTUD7B-depleted MDA-MB-231 cells, compared to control cells. G) Venn diagram showing the overlap in transcripts from (F) categorized by positively-regulated and negatively-regulated gene sets. H) Gene ontology analysis of transcripts negatively co-regulated by OTUD7B and LSD1 (FC > 1.5). I) Fold enrichment of normalized LSD1 and H3K4me2 ChIP-seq reads at enhancer regions of OLco-regulated transcripts. Negatively-regulated genes (n = 1308); positively regulated genes (n = 1120). *P* values were calculated by student's t test. J) Fold enrichment of normalized H3K4me2 ChIP-seq reads at promoter regions of OLco-regulated transcripts. *P* values were determined by student's t test. K) IB analysis of MDA-MB-231 cells infected with the indicated lentiviral constructs with the indicated antibodies.

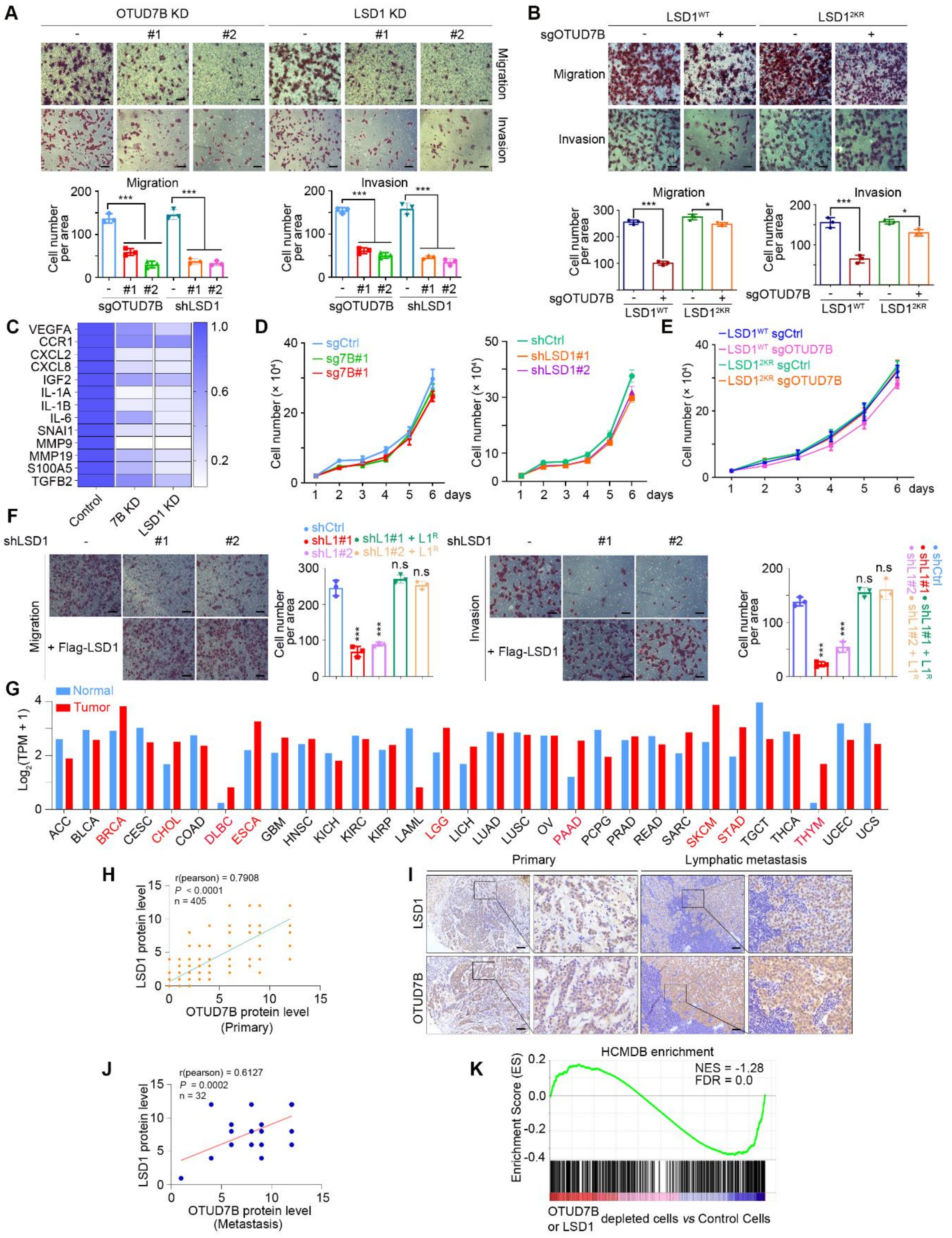

Figure S7. OTUD7B promotes metastasis via deubiquitinating LSD1. A) MDA-MB-231 cells expressing the indicated sgRNAs or shRNAs were subjected to Transwell migration or invasion assays. Scale bars, 50 μm. P values were determined by two-tailed unpaired t test. B) LSD1 reconstituting LM2 cells expressing the indicated sgRNAs were subjected to Transwell migration or invasion assays as indicated. Scale bars, 50 μm. ***P < 0.001. P values were

determined by two-tailed unpaired t test. C) Heatmap revealed fold change of indicated pro-metastasis genes upon OTUD7B or LSD1 depletion by RNA-seq analysis. (D-E) Parental (D) or LSD1 reconstituting (E) LM2 cells infected with lentiviruses encoding the indicated shRNAs or sgRNAs were subjected to cell proliferation assay. F) LM2 infected with lentivirus encoding the indicated constructs were subjected to Transwell migration or invasion assays as indicated. Scale bars, 50 μm. ***P < 0.001. P values were determined by two-tailed unpaired t test. G) Histogram shows the median mRNA expression profile of OTUD7B across the indicated tumor samples (red) and paired tissues (blue) using TCGA database. The cancer types with OTUD7B expression levels greater than 2-fold are highlighted in red. Data were presented as mean ± SEM of three independent experiments. H) Pearson's correlation coefficient is shown to indicate the correlation between LSD1 and OTUD7B protein levels based on (Figure 7D). P values were calculated by Fisher's exact test. I) Immunohistochemical staining of LSD1 and OTUD7B in paired primary and lymphatic metastatic (n = 27) breast cancer samples. Scale bar, 100 μm. J) Pearson's correlation coefficient is shown to indicate the correlation between LSD1 and OTUD7B protein levels based on (I). K) GSEA of pro-metastatic genes associated with breast cancer in HCMDB database compared with OLco-regulated gene sets.

Table S1. Summary of DUB screening results.

USP	Interaction	USP	Interaction
USP1	-	USP41	-
USP2A	-	USP43	-
USP2B	-	USP44	-
USP3	-	USP45	-
USP4	-	USP46	-
USP5S	-	USP48	-
USP5L	-	USP49	-
USP6	-	USP50	-
**USP7**	+/-	USP51	-
USP8	-	USP53	-
USP9X	-	USP54	-
USP9Y	-	CYLD	-
USP10	-	DUB3	-
USP11	-	USPL1	-
USP12	-	USP27X	-
USP13	-	**USP28**	++
USP14	-	USP29	-
USP15	-	USP30	-
USP16	-	USP31	-
USP18	-	USP32	-
USP19	-	USP33	-
USP20	-	USP34	-
USP21	-	USP35	-
**USP22**	++	USP36	-
USP24	-	USP37	-
USP25	-	USP38	-
USP26	-	USP39	-
USP47	-	USP40	-

OTU	Interaction
ALG13	-
YOD1	-
OTUD1	-
OTUD3	-
OTUD5	-
OTUD4	-
**OTUD6A**	+/-
OTUD6B	-
OTUD7A	-
**OTUD7B**	++
A20	-
OTULIN	-
OTULINL	-
OTUB1	-
OTUB2	-
VCPIP1	-
ZRANB1	-
PARP11	-

MJD	Interaction
ATXN3	-
ATXN3L	-
JOSD1	-
JOSD2	-
JOSD3	-

ZUP	Interaction
ZUP1	-

UCH	Interaction
UCHL1	-
UCHL3	-
UCHL5	-
BAP1	-

JAMM	Interaction
COPS5	-
COPS6	-
PSMD14	-
BRCC3	-
MYSM1	-
PSMD7	-
EIF3F	-
EIF3H	-
STAMBP	-
STAMBPL	-
MPND	-
PRPF8	-

Table S2. Case number across different cancer types for analyzing *LSD1* and *OTUD7B* mRNA expression profile

TCGA ID	***Cancer types***	***Tumor***	***Normal***
ACC	Adrenocortical carcinoma	77	128
BLCA	Bladder Urothelial Carcinoma	404	28
BRCA	Breast invasive carcinoma	1085	291
CESC	Cervical squamous cell carcinoma and endocervical adenocarcinoma	306	13
CHOL	Cholangiocarcinoma	36	9
COAD	Colon adenocarcinoma	275	349
DLBC	Lymphoid Neoplasm Diffuse Large B-cell Lymphoma	47	337
ESCA	Esophageal carcinoma	182	286
GBM	Glioblastoma multiforme	163	207
HNSC	Head and Neck squamous cell carcinoma	519	44
KICH	Kidney Chromophobe	66	53
KIRC	Kidney renal clear cell carcinoma	523	100
KIRP	Kidney renal papillary cell carcinoma	286	60
LAML	Acute Myeloid Leukemia	173	70
LGG	Brain Lower Grade Glioma	517	207
LIHC	Liver hepatocellular carcinoma	369	160
LUAD	Lung adenocarcinoma	483	347
LUSC	Lung squamous cell carcinoma	486	338
OV	Ovarian serous cystadenocarcinoma	426	88
PAAD	Pancreatic adenocarcinoma	179	171
PCPG	Pheochromocytoma and Paraganglioma	182	3
PRAD	Prostate adenocarcinoma	492	152
READ	Rectum adenocarcinoma	92	318
SARC	Sarcoma	262	2
SKCM	Skin Cutaneous Melanoma	461	558
STAD	Stomach adenocarcinoma	408	211
TGCT	Testicular Germ Cell Tumors	137	165
THCA	Thyroid carcinoma	512	337
THYM	Thymoma	118	339
UCEC	Uterine Corpus Endometrial Carcinoma	174	91
UCS	Uterine Carcinosarcoma	57	78
Total cases		9497	5360

TCGA PanCan 2018

Table S3. Case numbers of different cancer types utilized for analyzing genetic alteration of *LSD1*

Cancer types (TCGA PanCan 2018)	***Case number***
Uterine	529
Melanoma	444
Bladder	411
Cholangiocarcinoma	36
Lung squ	487
PCPG	178
Pancreas	184
DLBC	48
Ovarian	584
Colorectal	594
Stomach	440
Uterine CS	57
Cervical	297
Esophagus	182
Sarcoma	255
Lung adeno	566
Mesothelioma	87
Liver	372
pRCC	283
GBM	592
Thymoma	123
Prostate	494
LGG	514
Head & neck	523
Breast Invasive Carcinoma Breast	1084
Testicular germ cell	149
ccRCC	511
ACC	92
chromophobe RCC	65
AML	200
Thyroid	500
Uveal melanoma	80
Total cases	***10961***

Table S4. shRNA or sgRNA sequences in detail.

Target	Sequence (5'—3')
h. Ctrl sgRNA	GACCAGGATGGGCACCACCC
h. OTUD7B sgRNA#1	GCTGAGTCTGTTGGTAACGG
h. OTUD7B sgRNA#2	ATAGAAGCTGCAGTTCGGTT
h. Ctrl shRNA	GCAAAGAAGGCCACTACTATA
h. LSD1 shRNA#1	GCCACATTTCGCAAAGGAAAC
h. LSD1 shRNA#2	GCAGTTGTGGTTGGATAATCC
h. p62 shRNA	GCGGCTTCCAGGCGCACTACC
h. CoREST shRNA	GCATCATAACCTTTATCATGC
h. USP22 shRNA#1	GGAACACATGCTTCATGAACT
h. USP22 shRNA#2	AGTCTCAACAATGACAACAA
h. USP28 shRNA#1	GCTGCCAACAAGGAAGTATTA
h. USP28 shRNA#2	GCTGGAATTTCCTCAGATTAT

Table S5. Antibodies list

mouse monoclonal anti-LSD1 (Immunoblotting, immunofluorescence, IHC and immunoprecipitation)	CST	Cat# 4218
rabbit polyclonal anti-LSD1 (Immunoblotting, ChIP-seq and ChIP-qPCR)	BETHYL	Cat# A300-215A
rabbit polyclonal anti-OTUD7B (Immunoblotting, immunofluorescence, IHC and immunoprecipitation)	Proteintech	Cat# 16605-1-AP
mouse monoclonal anti-OTUD7B (immunoblotting)	Proteintech	Cat# 66276-1-Ig
rabbit polyclonal anti-CoREST (Immunoblotting)	CST	Cat# 14567
mouse monoclonal anti-H3K9me2 (ChIP-seq and ChIP-qPCR)	Abcam	Cat#1220
rabbit polyclonal anti-H3K4me2	Millipore	Cat# 07-030
rabbit polyclonal anti-HDAC1 (immunoblotting)	Proteintech	Cat# 10197-1-AP
rabbit polyclonal anti-HDAC2 (immunoblotting)	Proteintech	Cat# 12922-3-AP
rabbit polyclonal anti-USP22 (immunoblotting)	Proteintech	Cat# 55110-1-AP
rabbit polyclonal anti-USP28 (immunoblotting)	Proteintech	Cat# 17707-1-AP
rabbit polyclonal anti-Flag (immunoblotting)	Proteintech	Cat# 20543-1-AP
rat monoclonal anti-HA (immunoblotting)	Roche	Cat# 3F10
mouse monoclonal anti-Actin (immunoblotting)	Sigma	Cat# A1978
mouse monoclonal anti-Myc (immunoblotting)	CST	Cat# 2276
rabbit polyclonal anti-Myc (immunoblotting)	Proteintech	Cat# 16286-1-AP
mouse monoclonal anti-GFP (immunoblotting)	Proteintech	Cat# 66002-1-lg
rabbit polyclonal anti-p62 (immunoblotting)	Proteintech	Cat# 18420-1-AP
mouse monoclonal anti-Cyclin B (immunoblotting)	CST	Cat# 4135
mouse monoclonal anti-Cyclin E (immunoblotting)	CST	Cat# 4129

mouse monoclonal anti-Cyclin D1 (immunoblotting)	Proteintech	Cat# 60186-1-Ig
rabbit polyclonal anti-CDK6 (immunoblotting)	CST	Cat# 14052-1-AP
mouse monoclonal anti-Tubulin (immunoblotting)	Proteintech	Cat# 66240-1-lg
rabbit polyclonal anti-PKD1 (immunoblotting)	ABclonal	Cat# A14200
rabbit polyclonal anti-RIN1 (immunoblotting)	Proteintech	Cat# 16388-1-AP
rabbit polyclonal anti-RBBP8 (immunoblotting)	Proteintech	Cat# 12624-1-AP
rabbit polyclonal anti-Keap1 (immunoblotting)	Proteintech	Cat# 10503-2-AP
rabbit polyclonal anti-RASSF5 (immunoblotting)	SAB	Cat# 27727
rabbit monoclonal anti-Snail (immunoblotting)	CST	Cat# 3879
mouse monoclonal anti-His (immunoblotting)	Proteintech	Cat# 66005-1-Ig
rabbit polyclonal anti-GST (immunoblotting)	Proteintech	Cat# 10000-0-AP
mouse monoclonal anti-Lamin B1 (immunoblotting)	Proteintech	Cat# 66095-1-Ig
mouse monoclonal anti-GAPDH (immunoblotting)	Santa cruz	Cat# sc32233

Table S6. Primers for quantitative real-time PCR

Target	Forward primer (5’—3;)	Reverse primer (5’—3’)
Keap1	ATCGATGGCCACATCTATG	GATCCTTCGTGTCAGCATTG
Sox15	GGACAGGGAAGAGGCAACCT	GTTTGCAGTGGGAAGAGCCATA
RIN1	TCTTCCTGAAGCGAAGGAGC	CCTGGGCTGGCTTTTCTCTC
RASSF5	GGAGCATCTTCGAGCAGCC	GTGAATTTACAGTTAGTGCAGCG
ANAPC2	GCCACGTGCAAGGTTCTT	TCTGGGAAGTCTCGGACGAT
BMP2	TCCATGTGGACGCTCTTTCA	ACCATGGTCGACCTTTAGGAG
PKD1	CCTTCCTCACGTTCTCAGGC	GCCAGCACACCAGACTCTTA
RBBP8	GGAGCACTCTGTGTGTGCAA	ATGTGCTTTGGCCATTGGAG
Cyclin D1	GACCCCGCACGATTTCATTG	CATGGAGGGCGGATTGGAAA
CDK6	GTCTGATTACCTGCTCCGCGA	TTACATAGCCTCTGCCCAAGC
Snail	CGAGTGGTTCTTCTGCGCTA	GGGCTGCTGGAAGGTAAACT
Gapdh	TCGGAGTCAACGGATTTGGT	TGGAATTTGCCATGGGTGGA

Table S7. Primers for LSD1 ChIP-qPCR.

Target	Forward primer (5’—3;)	Reverse primer (5’—3’)
Keap1	CCTCCCACCTCAGCCTC	AATTAGCCGGGTGTGGTG
Sox15	CCCTGGGGCAAGAAAGGC	CACGGCCCAAGCACCTGC
RIN1	GTTAAGCGGCCTCAGTC	TGAGGCCAGGGGCAGATG
RASSF5	CTACTAACAAGGGGAAAG	CTGTCTGGTCATCCAGGC
ANAPC2	GGAGGCCCGCCCTCCGC	CCTCTATGCCTGGGGGC
BMP2	ATTATCCCAAACGTTTGAGC	GGTCTCCCATTTGGCTGGCG
PKD1	TTTTGAGACAGGGTCTTG	TGAGCTGTGATCACACCAC
RBBP8	GACCTGGCTGGAAAGCC	GGGACATTTCAGGGGTCC
Cyclin D1	GCCGCCAGGTATGCGGCTGC	GGGTGGTGCAGGGACCAGTC
CDK6	GATTATCACACGGCATCCTC	TGGGTGCGTTTTAGGGACAG
Snail	AGCCGGGCGGAGGAAATTTC C	CGGGACACCTGACCTTCCG

Table S8. Primers for H3K4me2 ChIP-qPCR.

Target	Forward primer (5'—3;)	Reverse primer (5'—3')
Keap1	CCCTTCTCACTGTCCCTTC	AGATGGTGGCGCGCAGC
Sox15	CACCACCCCAACCCCTTTC	CTCGGCCTTTCTTGCCCC
RIN1	GGGAGGAGAAGGATGTC	CGGGCCCTGCTCCTTG
RASSF5	CCTGAGCGGCCCCGAGC	GCGCCGGGACACAGAG
ANAPC2	TGGCATTCCAGAACTCAG	CCTGGAGGAGTGGTTCG
BMP2	GGAGCTAGCGCGGAGCGCCC	CCCGAGGGACGCGTGGCCCC
PKD1	TCCCAAACTGCTGGGATTC	ATTCAACAACAGACAGG
RBBP8	ATCCGCGTCCATACCCC	TGCAGGGAAGCATGTGTAG
Cyclin D1	GCCAGCCGCCCTGGTGG	GGTGCAGGGACCAGTCC
CDK6	GATTATCACACGGCATCC	GCCAACCTGAGACATGC

Table S9. Primers for H3K9me2 ChIP-qPCR.

Target	Forward primer (5'—3;)	Reverse primer (5'—3')
Keap1	CCCTTCTCACTGTCCCTTC	AGATGGTGGCGCGCAGC
Sox15	CACCACCCCAACCCCTTTC	CTCGGCCTTTCTTGCCCC
RIN1	GGGAGGAGAAGGATGTC	CGGGCCCTGCTCCTTG
RASSF5	CCTGAGCGGCCCCGAGC	GCGCCGGGACACAGAG
ANAPC2	GGAGGCCCGCCCTCCGC	CCTCTATGCCTGGGGGC
BMP2	GGAGCTAGCGCGGAGCGCCC	CCCGAGGGACGCGTGGCCCC
PKD1	TTTTGAGACAGGGTCTTG	TGAGCTGTGATCACACCAC
RBBP8	ATCCGCGTCCATACCCC	TGCAGGGAAGCATGTGTAG
Cyclin D1	CCGACCATCCGCCCAGG	CTGAAATCACCTTCACG
CDK6	CTGTCTCTGCTCTCTGTC	CCTTGTGGTGCTTGGCAG
Snail	ATTGCGGGCTCGGGAGAC	CTGCCCTCTACACGGCAC

Table S10. Gene sets utilized in GSEA analysis.

Gene Set Utilized	
**HALLMARK_EPITHELIAL MESENCHYMAL TRANSITION**	CD44, VCAN, DAB2, FOXC2, GJA1, CXCL1, IGFBP2, IL6, CXCL8, ITGAV, ITGB3, JUN, LOX, LOXL2, MMP1, MMP2, MMP3, MMP14, TNFRSF11B, SERPINE1, PLAUR, HTRA1, PTHLH, CXCL12, SNAI2, SPP1, TAGLN, TGFB1, TGM2, TIMP3, VCAM1, VEGFA, VEGFC, VIM, WNT5A, ADAM12, SLIT2, CADM1
**HALLMARK_TNFA SIGNALING VIA NFKB**	BIRC2, ATF3, BMP2, BTG1, CD44, CEBPB, KLF6, HBEGF, EGR1, F3, CXCL1, CXCL2, CXCL3, ICAM1, IL6, JUN, SMAD3, MYC, NFKB1, SERPINE1, PER1, PLAU, PLAUR, PTGS2, CCL5, STAT5A, TAP1, TNF, VEGFA, FOSL1, IRS2, SPHK1, KLF4, TRIP10, ACKR3
**HALLMARK_IL6 JAK STAT3 SIGNALING**	BAK1, CBL, CD44, CXCL1, CXCL3, HMOX1, IL2RG, IL6, ITGB3, JUN, LEPR, STAT1, STAT3, TGFB1, TNF, TYK2
**HALLMARK_TGF BETA SIGNALING**	XIAP, RHOA, BMP2, CDH1, CTNNB1, ID1, SMAD3, SERPINE1, SKIL, TGFB1, ARID4B
**GO_Positive Regulation of Cell Motility**	AAMP, ACTN4, AKT1, AKT2, ANXA1, ANXA3, ARF6, RHOA, RHOC, BMP2, BMP4, BMP7, C1QBP, CAV1, CD74, CD151, CCR7, DAB2, DOCK1, HBEGF, EGFR, F2R, F3, PTK2B, FER, FGFR1, VEGFD, FOXC2, FLT4, GATA3, GCNT2, GRN, HGF, HIF1A, HMGB1, HMOX1, HSPA5, HSPB1, ICAM1, IGF1R, IGFBP5, IL6, CXCL8, ITGA6, ITGAV, ITGB3, KDR, LGALS3, LYN, SMAD3, MDK, MMP9, MMP14, NEDD9, DDR2, SERPINE1, PAK1, PDGFB, PFN1, PIK3CG, PLAU, MAPK1, MAPK3, PTGS2, PTK2, RAC1, RAC2, RET, RPS6KB1, S100A7, CCL5, SDCBP, CXCL12, SELE, SNAI2, SNAI1, SOX9, SP1, SRC, STAT3, STAT5A, TAC1, TACR1, TERT, TGFB1, TGFBR2, TNF, TWIST1, VEGFA, VEGFC, WNT5A, CXCR4, IRS2, FADD, NRP2, CCN4, SPHK1, CLDN1, ARHGEF2, CXCL14, BCAR1, IQSEC1, AKT3, GPNMB, SEMA4D, ACKR3, RAB25, PREX1, FERMT3, STK11, SLIT2, ANGPT2, ATP6V1C1, AXL, BMP5, CBL, CBLB, CDC42, EFNB1, ERBB2, ERBB4, EFEMP1, FGFR4, FOXC1, FOXO1, GSK3B, NRG1, IGFBP2, LEP, LOX, MMP2, MST1, MST1R, NTRK1, ENPP1, PIK3CA, PLAUR, PRL, PRLR, PTEN, PTPN3, PXN, ROBO1, SDC2, SHC1, SYK, TSC2, VAV2, YES1, WASL, ADIPOQ, ELMO1, VAV3, AGR2, TXNIP, GIT1, CCDC88A, EPHA10, XIAP, BMP6, RUNX2, CCNA2, CD44, CREB1, CTNNB1, E2F1, EGR1, GDF2, IBSP, ID1, IL10, JUN, SMAD4, MYC, PPARG, HTRA1, SKIL, CLDN5, TP53, VCAM1, KLF4, PPARGC1A, F11R, DLL4, HTRA3

**GO_Chemotaxis**	AGTR1, ALCAM, ANGPT2, ANXA1, RHOA, BMP4, BMP7, BSG, C1QBP, CD74, CCR5, CCR7, CREB1, HBEGF, EFNB1, ERBB2, F3, PTK2B, FER, FGFR1, VEGFD, GATA3, CXCL1, CXCL2, CXCL3, HGF, NRG1, HMGB1, HOXB9, HSPB1, IL6, CXCL8, IL10, ITGAV, ITGB3, KDR, L1CAM, LGALS3, LOX, LYN, SMAD3, SMAD4, MDK, MIF, MST1, NOTCH3, NTRK1, SERPINE1, PDGFB, PIK3CA, PIK3CG, PLAU, PLAUR, MAPK1, MAPK3, PTK2, RAC1, RAC2, RET, ROBO1, S100A7, S100A8, S100A9, SCN1B, CCL5, CCL14, CCL18, CXCL12, SHC1, SRC, SYK, TGFB1, TSC2, VCAM1, VEGFA, VEGFC, EZR, VLDLR, WNT5A, CXCR4, FOSL1, TNFSF11, IRS2, TNFRSF11A, NRP2, SLIT2, CXCL14, BCAR1, VAV3, GPNMB, SEMA4D, EVL, ENAH, ACKR3, PREX1, WNT3A, EPHA10, AKT1, BMP5, CAV1, CD44, CDC42, CEACAM5, HMOX1, ICAM1, ITGA6, LEP, EPCAM, MMP1, MMP9, MMP14, CD200, SDC2, SELE, TNF, YES1, FADD, WASL, F11R
**GO_Regulation of Cell Adhesion**	ACTN4, AKT1, ANGPT2, ANXA1, RHOA, ARHGDIA, ARHGDIB, BMP2, BMP4, BMP6, BMP7, C1QBP, CAV1, CBLB, CD44, CD74, CDC42, CDH1, CDKN2A, CEBPB, CCR7, DOCK1, EFNB1, ERBB2, PTK2B, FOXC2, GATA3, GCNT2, GSK3B, HMGB1, IBSP, ICAM1, IGFBP2, IL6, CXCL8, IL10, ILK, IDO1, IRAK1, ITGA6, ITGAV, JAK1, KDR, LEP, LGALS3, LYN, EPCAM, SMAD3, MDK, MMP14, MUC1, MYB, NEDD9, SERPINE1, PAK1, PDGFB, PIK3CA, PIK3CG, PLAU, PLAUR, PTEN, PTK2, RAC1, RAC2, RAC3, RET, CCL5, CXCL12, SELE, SLC9A1, SNAI2, SOX9, SRC, SYK, TGFB1, TGFBR2, TGM2, TNF, UTRN, VCAM1, VEGFA, WNT5A, YES1, CXCR4, TNFSF11, FADD, DNAJA3, KLF4, ADIPOQ, DLC1, VAV3, GPNMB, SEMA4D, AGR2, F11R, FOXP3, PREX1, PEAK1, CD276, FERMT3, WNT3A, S100A8, S100A9, EZR, AHR, AXL, BAK1, BST2, CD151, KLF6, EGR1, EZH2, LEPR, MIF, NTRK1, PRKDC, RAG2, STAT3, STK11, TAC1, TLR4, TP53, TNFRSF4, IRS2, CADM1, TLR9, DLL4, FER, GRN, HMOX1, IL13RA2, MMP8, CD200, SPHK1, IL1RL1, ADGRF5, CRP, ACE, MAPK1, MAPK3
**GO_Vasculature Development**	AAMP, ADM, AGTR1, AHR, AKT1, ANGPT2, ANXA1, ANXA2, ANXA3, RHOA, BAK1, BMP4, BMP7, BRCA1, BSG, BTG1, CAV1, CDC42, CTNNB1, EGR1, ERBB2, F3, PTK2B, VEGFD, FOXC1, FOXC2, FLT4, GDF2, GRN, HGF, HIF1A, HK2, HMGB1, HMOX1, HSPB1, ID1, IL6, CXCL8, IL10, ITGAV, ITGB3, JAK1, JUN, KDR, LEP, LEPR, LOX, LOXL2, MDK, MMP2, MMP14, NFATC1,

	SERPINE1, PDGFB, PIK3CA, PIK3CG, PPARG, MAPK1, PRL, PTEN, PTGS2, PTK2, PTK7, ROBO1, S100A7, SHC1, SIX1, SP1, STAT1, STK11, SYK, TERT, TGFB1, TGFBR2, CLDN5, TNF, TWIST1, VAV2, VEGFA, VEGFC, WNT5A, CXCR4, ADAM12, FOSL1, HMGA2, RECK, NRP2, SPHK1, KLF4, SLIT2, AKT3, PAK4, YAP1, VAV3, GPNMB, HTATIP2, DLL4, FOXJ2, ACKR3, SOX17, ADGRF5, BMP2, BMP5, BMP6, BRCA2, CEBPB, EGFR, ESR1, FGFR1, GATA3, GJA1, GLI1, SFN, IGFBP5, SMAD3, MST1, MYC, NME1, PGR, SERPINB5, PRKDC, HTRA1, RB1, CXCL12, SNAI2, SOX9, STAT5A, TLR4, TNFSF11, CLDN1, ODAM, WNT3A, RICTOR, ARF6, ARHGDIB, C1QBP, DOCK1, HBEGF, EFNB1, ERBB4, FER, ITGB4, MMP9, DDR2, PAK1, PFN1, PXN, RAC1, RET, SRC, TAC1, PDLIM1, MTA2, BCAR1, IQSEC1, SEMA4D, PTP4A3, EVL, RAB25
